# A fully 3D-printed versatile tumor-on-a-chip allows multi-drug screening and correlation with clinical outcomes for personalized medicine

**DOI:** 10.1038/s42003-023-05531-5

**Published:** 2023-11-13

**Authors:** Eliana Steinberg, Roy Friedman, Yoel Goldstein, Nethanel Friedman, Ofer Beharier, Jonathan Abraham Demma, Gideon Zamir, Ayala Hubert, Ofra Benny

**Affiliations:** 1 The Institute for Drug Research, The School of Pharmacy, Faculty of Medicine, The Hebrew University of Jerusalem, Jerusalem, Israel; 2 School of Computer Science and Engineering, Center for Interdisciplinary Data Research, The Hebrew University of Jerusalem, Jerusalem, Israel; 3 Hadassah Medical Center and The Hebrew University of Jerusalem, Jerusalem, Israel; 4 Department of General Surgery, Hadassah Medical Center and Faculty of Medicine, The Hebrew University of Jerusalem, Jerusalem, Israel; 5 Oncology Department, Hadassah Medical Center, Jerusalem, Israel

**Keywords:** Lab-on-a-chip, Cancer models

## Abstract

Optimal clinical outcomes in cancer treatments could be achieved through the development of reliable, precise ex vivo tumor models that function as drug screening platforms for patient-targeted therapies. Microfluidic tumor-on-chip technology is emerging as a preferred tool since it enables the complex set-ups and recapitulation of the physiologically relevant physical microenvironment of tumors. In order to overcome the common hindrances encountered while using this technology, a fully 3D-printed device was developed that sustains patient-derived multicellular spheroids long enough to conduct multiple drug screening tests. This tool is both cost effective and possesses four necessary characteristics of effective microfluidic devices: transparency, biocompatibility, versatility, and sample accessibility. Compelling correlations which demonstrate a clinical proof of concept were found after testing and comparing different chemotherapies on tumor spheroids, derived from ten patients, to their clinical outcomes. This platform offers a potential solution for personalized medicine by functioning as a predictive drug-performance tool.

## Abstract (web summary)

A unique 3D-printed device made of sustainable materials was developed to produce 3D spheroids from primary cells and perform drug screening assays in a biomimetic environment.

## Introduction

It is well established that tumors display substantial intratumor, intertumor, intrapatient and interpatient heterogeneity, making the “one-size-fits-all” conventional treatment approach insufficiently effective in combating cancer^1,2^. Even patients diagnosed with the same kind of cancer may present different tumor phenotypes and respond differently to the same treatment^3,4^. Due to the many complexities of cancer, the development of reliable tumor tissue culture models that can mimic a range of malignancy behaviors more accurately would be of great value in the battle against this disease. Such models could also be clinically relevant as predictive drug-performance tools, enabling physicians to replace the practice of “trial and error” for treatment selection with rational selection based on ex vivo models.

Widely used two-dimensional (2D) cell cultures lack key features that are critical for replicating physiological systems^5^, such as spatial cell-cell interactions, extra-cellular matrices (ECM)^6^, dynamic metabolic demand, increased hypoxia due to mass growth^7^, and effects of the tumor microenvironment (TME)^8^. 2D-culture inaccuracies in cytotoxicity assays can lead to misinterpretation and poor prediction of in vivo behavior. For example, tissue processes such as hypoxia are known to contribute to treatment resistance^9^. Drug screening in monolayer models that have been the main drug selection tool for years may be partly responsible for the high rate of clinical trial failures for new treatment molecules^10^.

To that end, 3D cellular models are being developed and studied extensively^11–14^. One of the most promising 3D cellular models is the multicellular spheroid tumor model^15–17^. Spheroids are ex vivo cellular aggregate *“*micro-tissues” that exhibit tissue-like metabolic activity governed by nutrient and oxygen diffusion mechanisms similar to tumors^18,19^. These conditions are similar to hypoxic micro-tumors in vivo that are known to negatively affect a tumor’s sensitivity to anticancer drugs and to contribute to its acquired resistance^20,21^.

However, despite the many advantages of 3D cultures, the lack of continuous fluid flow in their surroundings results in their failure to capture the real complexity of tissues. For example, fluid flow subjects tissues to mechanical forces generated by fluid shear stress, hydrostatic pressure and tissue deformation that can substantially influence cancer cell behavior^22–25^. Animal models replicate the in vivo TME complexity more closely than 2D culture models, however, they raise ethical concerns and are not always a good representation of human pathophysiology. The shift toward human tissue models of high physiological mimicry substantially reduces the high costs associated with the use of animals and solves many ethical issues^26–29^. In this regard, organ-on-a-chip platforms open important possibilities that can become the future state of the art, especially in light of the Food and Drug Administration’s recent announcement regarding the acceptance of animal alternatives in the track of drug approvals^30^.

Meeting these needs, tumor-on-a-chip technologies enable the recapitulation of the physiologically relevant physical microenvironment of cancers while sustaining fluid perfusion in vitro^31–33^. The standard approach to fabricating microfluidic devices is based on soft lithography techniques mainly using polydimethylsiloxane (PDMS)^34,35^. This is due to its favorable properties of biocompatibility, optical transparency, and gas permeability. However, its tendency to bind or adsorb small hydrophobic molecules^36,37^ make it less suitable for drug-based studies since it may change target concentrations and result in drug delivery to undesired regions in the microfluidic device^38,39^. In addition, its lack of durability for lengthy experiments, its requirement of a cleanroom setup for fabrication and requisite extensive manual procedures has led to the increasing use of stereolithography (SLA) 3D printing as an alternative^40^. In this fabrication method, 3D structures are built in a layer-by-layer photopolymerization deposition with a UV-curable liquid resin^41^.

Printing in 3D with photocurable resins enables versatility and complexity in device designs that are often impossible or very difficult to achieve otherwise. Moreover, 3D printing considerably reduces the post-processing time and costs^32^. However, despite the obvious benefits, a substantial obstacle to applying 3D printing to cell culture studies is that SLA resins are not sufficiently biocompatible and their limited optical transparency limits sample visualization. Most of the commercially available photocurable resins have proprietary formulations with scarce information regarding their composition and cytocompatibility^42,43^.

In this work, we present a technological platform involving a fully 3D-printed microfluidic device that maintains patient-derived multicellular spheroids for long periods of time, enabling multiple drug screening tests for personalized therapy purposes. To accomplish this, we developed a protocol for 3D-printing commercially available photocurable resins directly onto glass slides with a post-cleaning process that ensures biocompatibility. Data from cell culture assays demonstrated the biocompatibility of these devices over a 7-day period. Two main designs were developed, demonstrating the versatility of the platform, the optical transparency enabling continual visualization, and the capability to physically access biological samples without disturbing the course of the experiment. To demonstrate the clinical benefits of our fabricated devices, we assembled spheroids using tumor samples taken from ten different patients who were each treated with different chemotherapies recommended by their oncologists and whose ex vivo outcomes were compared with their clinical results. Compelling correlations were observed, including a significant reduction in viability of Oxaliplatin-treated spheroids comprised of cells from patients carrying the BRCA2 mutation, which is known to confer high sensitivity to platinum drugs^44^. Interestingly, four of the chemotherapy treatments induced a positive trend, where spheroid size shrinkage corresponded with viability reduction, while with two chemotherapies this trend was frequently reversed, indicating the importance of combining size-based analysis with viability assays in drug screening tests. Altogether our results suggest the potential for using this microfluidic system as a reliable tool for predicting the optimal drug treatment for each patient.

## Results

### 3D printing directly onto glass slides for optical transparency

**Fig. 1:**
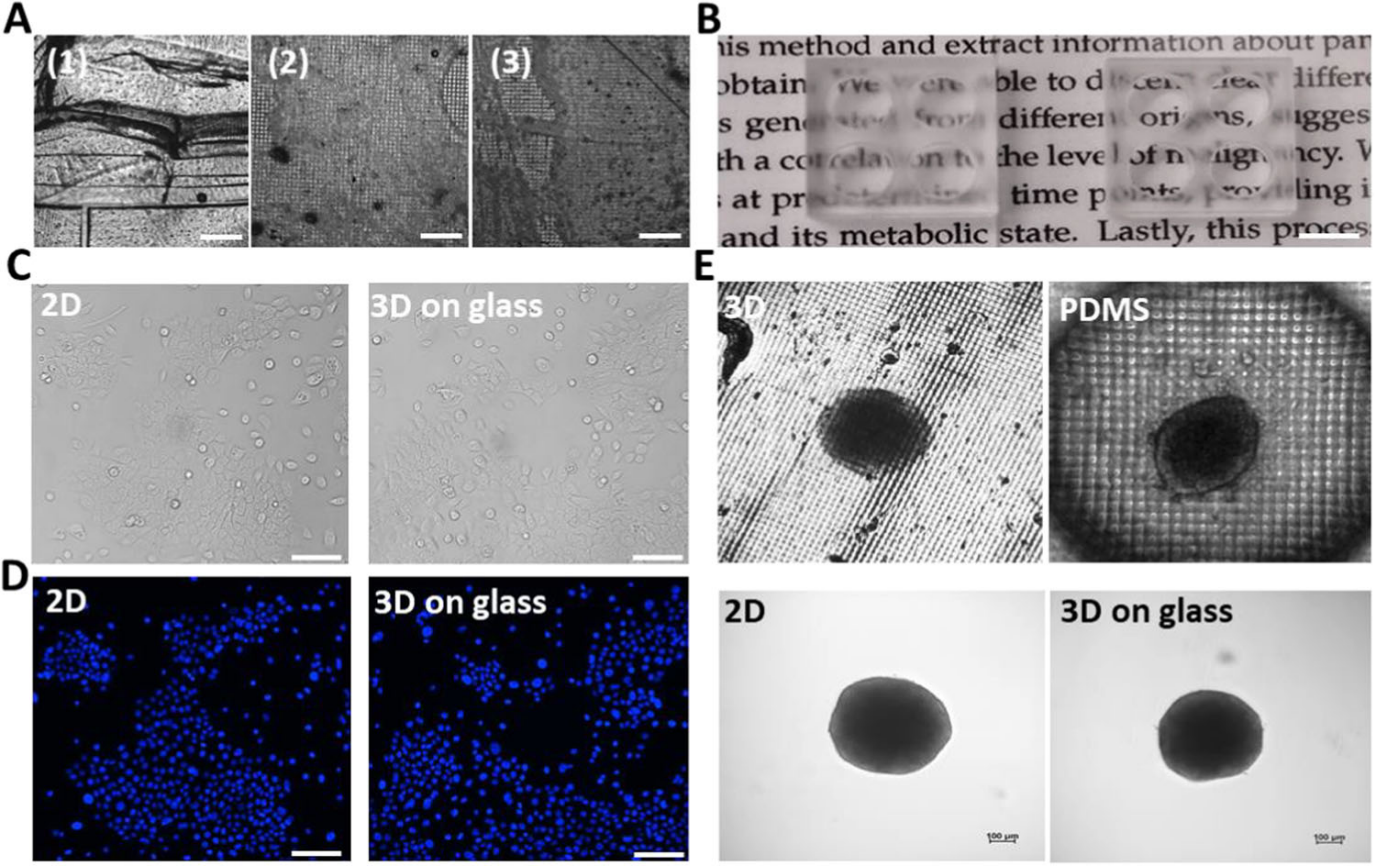
3D-printing onto glass slides improves optical transparency. **A** Comparing the transparency of 3D-printed resins (1) BV-007A, (2) Luxaprint and (3) Freeprint used to print 4-well molds designed with a well diameter of a standard 96-well plate (radius = 3.19 mm). The mold bottoms of 2 mm thickness cracked after 72 h in a 37 °C incubator. Scale bar = 500 um. **B** Comparing the transparency of a 4-well Freeprint mold (radius = 3.19 mm, left) with a 4-well Freeprint mold printed straight onto a glass slide (well bottom is glass, right). Scale bar = 6 mm. **C** BxPC-3 were imaged 24 h after seeding (50,000 cells/well) in a standard 48-well plate (control, 2D) and in a 3D-printed Freeprint well printed onto a 1 mm glass slide coated with PLL. Scale bar = 100 um. **D** BxPC-3 cells were stained with Hoechst and imaged 24 h after seeding. Scale bar = 100 um. **E** Images obtained 48 h after transfer of BxPC-3 spheroids to Freeprint 3D-printed mold, PDMS, standard low-attachment surface polystyrene 12-well plate (2D) and Freeprint mold printed onto a glass slide. Scale bar = 100 um.

To overcome the drawbacks of currently used silicon-based materials to fabricate microfluidic devices for cell culturing, we worked on developing a one-step method for 3D printing similar devices. Figure 1a shows images of molds printed from three different resins. The molds contain wells with the same dimensions as a standard 96-well plate (radius of 3.19 mm). The well bottoms consist of a thin 2 mm layer, allowing maximal optical transparency while avoiding breakability. The molds were placed in a 37 °C incubator and imaged after 72 h. In all cases the wells’ bottom surface did not appear to be smooth and the transparency was poor. This may be attributed to the fact that the resins were not transparent enough since the resolution of the voxelized models resulted in a crisscross grid. In addition, the printed resins peeled and cracked over time. Therefore, our next step was to develop a protocol for activating glass slides to be used as the base bottom of the 3D-printed devices (Fig. 1b), thus accomplishing maximal optical transparency. After surface treatment and heating, the glass slides were mounted onto the printer platform providing the base for printing. The molds printed onto glass slides were then cured using UV light for 1 h. Images of BxPC-3 cells and spheroids seeded in standard polystyrene dish plates and 3D-printed molds on glass are presented in Fig. 1c-e for comparison. The optical transparency of monolayer cells and 3D-cell aggregates seeded in devices printed onto glass slides is much higher, as imaged by brightfield and fluorescent microscopes. Unlike the glass-bottom devices, the image resolution of BxPC-3 spheroids placed in printed resin molds or PDMS was poor (Fig. 1e) due to the uneven surface topography.

### Biocompatibility of 3D-printed molds in long-term culture of cells and multicellular spheroids

**Fig. 2:**
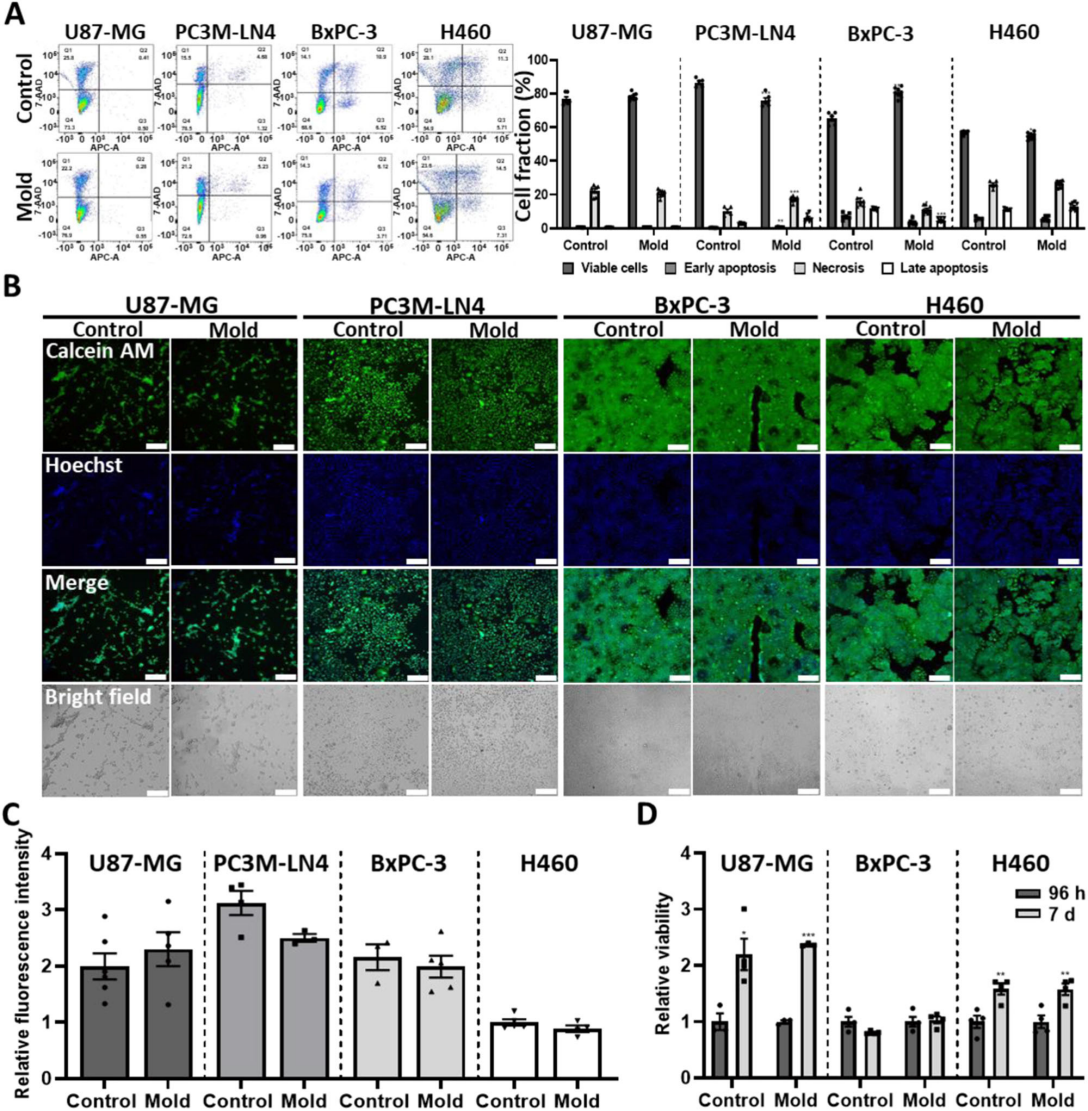
3D-printed molds are biocompatible for long term culture of cells and spheroids. **A** U87-MG, PC3M-LN4, BxPC-3 and H460 cells were seeded (100,000 cells/well) in standard 12-well plates (control) and 3D-printed molds containing the same well diameter as the wells in the 12-well plates (radius = 17.7 mm). After 96 h of incubation, the cells were stained with 7-AAD/Annexin and FACS was performed using the LSRFortessa Flow Cytometer. Cell fraction in the different stages of the cell cycle is similar between cells seeded in control wells and 3D-printed mold wells. *n* = 8. **B** U87-MG, PC3M-LN4, BxPC-3 and H460 cells seeded in 17.7 mm radius wells were stained with Hoechst and Calcein AM 96 h after seeding and imaged. Scale bar = 200 um. **C** Calcein AM fluorescent intensity was normalized to Hoechst fluorescent intensity. No statistical differences between the cells grown in the control plate and the mold were detected. *n* = 6. **D** U87-MG, BxPC-3 and H460 cells were seeded at 5000 cells/microwell in agarose microwells and were transferred after 24 h to 3.19 mm radius wells. A WST1 viability assay was performed on U87-MG, BxPC-3 and H460 spheroids 96 h and 7 d after their transfer to standard 96-well plates and 3D-printed molds containing the same well diameter. Spheroids’ viability in the mold condition was compared to spheroids’ viability in the control condition of the same time point. Similar trends in spheroid viability were observed under both conditions. *n* = 4. ***p < 0.001, **p < 0.01 and *p < 0.05 compared with control cells. Results are presented as mean ± SEM.

Photocurable resins are widely considered to be toxic to cells, however the degree of these materials’ cytotoxicity remains largely unclear^45^. We therefore opted to investigate the biocompatibility of the 3D-printed resins, given our specific procedures, for cell and spheroid culturing using various assays. Fig. S1 reveals the cytotoxicity of three different commercial resins used for printing molds for cell cultures. Fig. S2a shows that when using our protocol, there is no significant difference in the viability or proliferation of U87-MG, PC3M-LN4, BxPC-3 and H460 cells, 24 h and 96 h after their seeding in standard 96-well plates or 3D-printed molds on glass slides having the same dimensions. Flow cytometry analyses of apoptosis and necrosis using Annexin-V-APC and 7-AAD staining of U87-MG, PC3M-LN4, BxPC-3 and H460 cells 96 h after seeding revealed no significant difference in the cell fraction of U87-MG cells in the different cell cycle stages, while PC3M-LN4 cells showed no more than a 10% reduction in the fraction of viable cells seeded in 3D-printed molds compared with control standard wells (Fig. 2a). In contrast, BxPC-3 cells cultured in 3D-printed molds showed an increase of 15% in the fraction of viable cells compared with standard control wells. H460 cells displayed less than a 3% difference in the fraction of viable cells between the two conditions. The flow cytometry results were further supported by cell staining with Calcein AM and Hoechst. Calcein AM fluorescent intensity was normalized to the fluorescent intensity of Hoechst nuclei staining and revealed no significant difference in the viability (Fig. 2b, c). Further validation of cell viability was done by monitoring U87-MG, BxPC-3 and H460 spheroids with Calcein AM 96 h and 7 d after their transfer to either a 96-well standard plate or its equivalent 3D-printed mold. Fig. S2b indicates similar trends in spheroid viability under both conditions over time. Spheroid viability was also quantified 7 d after their transfer using a WST1 reagent (Fig. 2d). U87-MG and H460 spheroids displayed similar increases in viability over time under both conditions, as opposed to BxPC-3 spheroids which showed the same levels of viability over time in both conditions. Taken together, the 3D-printed resins post-cleaned with our procedure displayed biocompatibility and suitability for the long term culture of cells and spheroids when compared to standard polystyrene culturing conditions.

### Spheroid culturing in microfluidic devices using the hanging drop technique

**Fig. 3:**
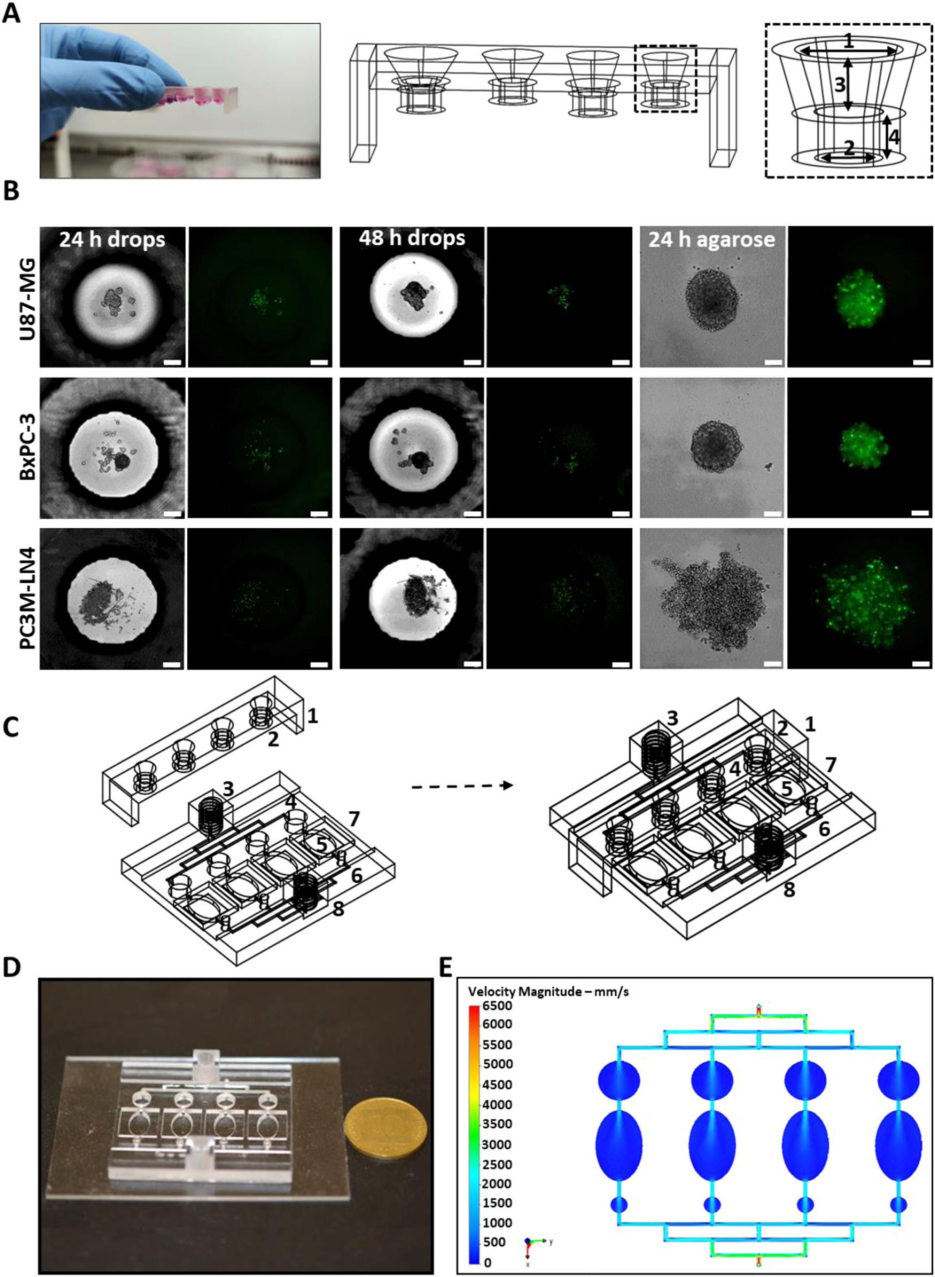
Spheroid culture in microfluidic device using the hanging drop method. **A** A 3D-printed mold containing four different hanging-drop well geometries. A shaking test showed that the well geometry on the far right (boxed) produced the most stable drop. This geometry enabled the formation of drops that remained stable even after 5 days in a humidified 37 °C incubator with no need to replenish the culture media. The well dimensions were as follows: (1) and (2) width of 5 and 2 mm respectively; (3) and (4) height of 2.88 and 1.92 mm respectively. **B** Bright-field and fluorescent images of U87-MG, PC3M-LN4 and BxPC-3 cells stained with CellTrace^TM^ CFSE and seeded at 8000 cells/60 uL drop. Cells were imaged 24 and 48 h after seeding and then transferred to 96-well plates coated with 2% agarose. They were imaged again 24 h after their transfer, exhibiting viable and spherical spheroids (obtained within 3 days of initial seeding). Scale bar = 300 um. **C** AutoCad® design displaying the convenient transfer of spheroids from the hanging drop mold. (1) Hanging drop mold, (2) hanging drop well, (3) “nut” inlet for flow, (4) inlet for the hanging drop, (5) spheroid chamber, (6) outlet for excess medium, (7) glass cover slide sealing spheroid chamber and (8) “nut” outlet for flow. **D** Fully 3D-printed device printed onto a glass slide. Coin radius = 1.1 cm. **E** Top view of simulated flow velocity profile of the full microfluidic device using a perfusion rate of 10 cm^3^/min.

One of the main methods used to create spheroids is the hanging drop technique^46^. For this, a mold containing hollow wells of four different geometries (variation in the diameter of the higher and lower chambers) was designed and 3D printed. Culture media was placed in each well and a “shaking test” was conducted as shown in Movie S1 and Fig. S3. An optimal well geometry was identified based on the ability to maintain the most stable drops under shaking (Fig. 3a). In this device, the drops lasted for 5 days in a humidified 37 °C incubator without needing media replenishment. To prove that the geometry of the device supported the formation of viable spheroids in stable drops, U87-MG, PC3M-LN4 and BxPC-3 cells stained with CellTrace^TM^ CFSE were seeded and monitored. Viable spheroids were formed 3 days after their initial seeding, as indicated by the green-fluorescent images (Fig. 3b). A microfluidic chip was designed to enable the convenient transfer of the drops containing the spheroids into a hosting chamber. Each drop was transferred individually into a separate compartment in the chip for the purpose of long-term culturing under flow conditions (Fig. 3c, d). It has been shown that shear stress, induced by liquid flow, influences the aggressiveness of tumor cells^25^. To ensure proper fluid behavior inside the microfluidic chip, CFD simulation was performed. Figure 3e shows the flow velocity simulation indicating laminar flow profiles. The flow velocity decreased in the relatively large geometrical culture compartments and increased again at the narrow outlet channels.

### 3D-printed “nut and bolt” chip for spheroid culture using the ultra-low attachment surface method

**Fig. 4:**
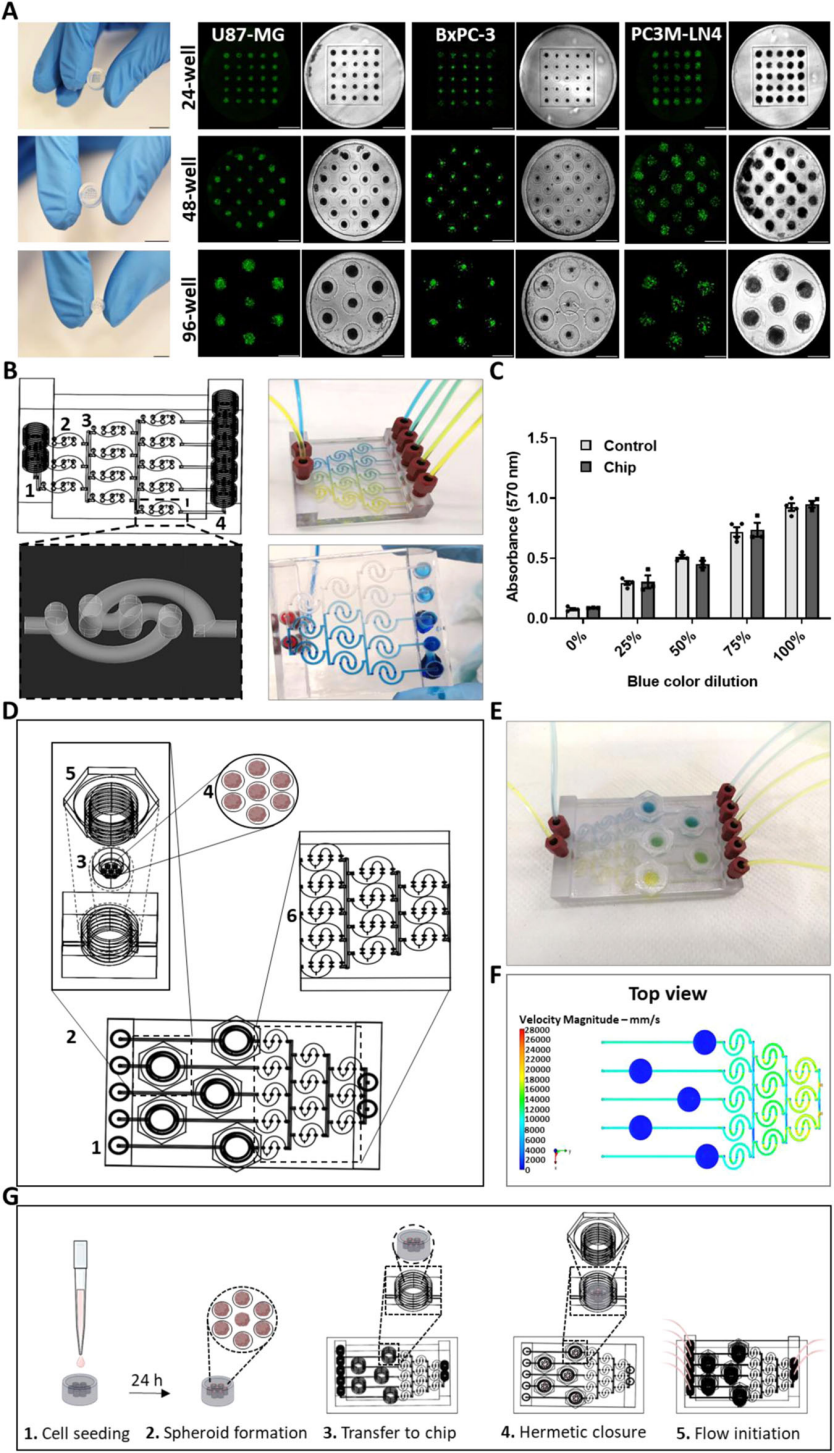
3D-printed “nut and bolt” chip for spheroid culture using the ultra-low attachment microwell method. **A** UltraPure^TM^ Agarose hydrogel microwells were fabricated by casting into three designs of 3D-printed complementary templates: 7, 21 and 25 microwells. U87-MG, BxPC-3 and PC3M-LN4 were seeded at 4000 cells/microwell immediately after their staining with Cell Proliferation Staining Reagent Green Fluorescence Cytopainter and imaged after 24 h. Scale bar of molds from top to bottom: 12.4, 9.83 and 5.5 mm respectively. The scale bar of microscopic images is 0.8 mm. **B** AutoCad® design and 3D-print of chip designed to mix and create a concentration gradient. Channels were designed in a spiral shape extending over several planes to achieve maximum mixing in minimal horizontal space. Right top and bottom—blue and yellow food coloring, and blue food coloring and DDW were flowed through the chip respectively. (1) “Nut” inlet for flow, (2) spiral mixing channel, (3) gradient channel and (4) “nut” outlet for flow. **C** Blue food coloring and DDW flowed simultaneously through the chip and collected at the outlets for absorbance reading at 570 nm. *n* = 4. All results were compared to manually prepared dilutions of the blue food coloring and DDW and were not statistically different. Results are presented as mean ± SEM. Five concentrations were collected at the outlets: 0% blue coloring (100% DDW), 25% blue coloring (75% DDW), 50% blue coloring (50% DDW), 75% blue coloring (25% DDW) and 100% blue coloring (0% DDW). **D** AutoCad® design of “nut and bolt” chip. (1) Illustration of full concentration gradient microfluidic device used for spheroid culture, (2) spheroid culture chamber in the shape of a “nut”, (3) mold containing spheroids, (4) microwells with spheroids, (5) “bolt” used to seal the culture chamber and (6) mixing and gradient concentration-generating section of the device. **E** Fully 3D-printed chip for spheroid culture. Blue and yellow food coloring flowed simultaneously through the chip, producing a color gradient. **F** Top view of a simulated flow velocity profile of the full microfluidic device using a perfusion rate of 10 cm^3^/min. **G** Illustration of the operating steps for using the “nut and bolt” chip for spheroid culture. (1) Cell seeding into agarose mold containing microwells, (2) spheroids are formed after 24 h, (3) molds containing the spheroids are transferred to the culture chambers in the device, (4) 3D-printed “bolts” are used to seal the chambers, (5) mixing and gradient concentration is generated once flow is initiated. *Created with permission from Biorender.com.

A common method for creating spheroids is the use of ultra-low attachment hydrogel surfaces^47^. For this approach, templates containing the complementary geometry of the microwells were designed in three different variations to produce 7, 21 and 25 microwells (Fig. S4a). The agarose hydrogel arrays were fabricated as the complement to the printed molds and placed in 96, 48 and 24-well standard plates respectively. U87-MG, BxPC-3 and PC3M-LN4 cells were stained with Cell Proliferation Staining Reagent Green Fluorescence Cytopainter and seeded at 4,000 cells/microwell. The wells were imaged 24 h after cell seeding, showing the formation of homogenous viable spheroids (Fig. 4a). To produce a concentration gradient flow on a chip, spiral channels extending over several planes were designed to maximize mixing in a minimal horizontal area (Fig. S4b). To demonstrate this, blue and yellow food coloring were flowed simultaneously through the chip, showing the formation of a color gradient (Fig. 4b). Figure. 4c shows the optical absorbance at 570 nm which was obtained by diluting the blue food coloring with DDW after its collection from the device’s outlets. The gradient generated on the chip was similar to that generated by a standard curve. Figure. 4d illustrates the incorporation of the optimized gradient compartment into the design of the complete “nut and bolt” microfluidic chip. The full chip design shows the chambers intended for spheroid culture in the shape of a “nut”, allowing repeated opening and closing of the chamber and convenient access to biological samples after removing the “bolt”, while providing a completely sealed and leakproof device when the bolt is replaced. The mold containing several microwells is placed in each culture chamber, thus enabling the growth of numerous spheroids under various conditions. Blue and yellow food coloring were perfused into the chip, confirming the generation of a concentration gradient to which the culture chambers were exposed (Fig. 4e). A flow velocity simulation was performed, showing a laminar flow profile in the full microfluidic device (Fig. 4f and S4d). As indicated, the flow velocity rose and fell alternately in the spiral channels extending over several planes, thus accomplishing effective mixing. The simple operating steps for using the “nut and bolt” chip for spheroid culture are illustrated in Fig. 4g. Figure S4c depicts a simplified prototype of the “nut and bolt” device without the concentration gradient forming section.

### Responses of patient-derived multicellular tumor spheroids to chemotherapy

To confirm the use of the ex vivo culture device for therapy personalization, we collected tumor samples from ten patients (referred to as T1-T10 and described in the Methods section). Table 1 details patient profiles and their responses to therapies. To provide a proof of concept for the validity of our approach in predicting responses to drugs, each fresh tissue was processed, and patient-derived 3D multicellular tumor spheroids were formed and treated with different chemotherapy combinations. Since it is difficult to correlate patient treatment regimens with ex vivo experiments, the chemotherapy concentrations were established according to IC50 calculations (Fig. S5). The reduction in their viability is reported in Fig. S6a. The viability of Oxaliplatin-treated spheroids comprised of T1 cells from a patient carrying a BRCA2 mutation was reduced by ~90%. Similar reduction in viability was observed in the T4 and T5 spheroids obtained from two patients carrying similar mutations (TP53, KRAS, PIK3K and SMAD4). Oxaliplatin induced a ~ 56% reduction in viability, 5-FU a ~ 45% reduction, while Gemcitabine induced only a mild reduction in viability.

**Table 1:** Patient characteristics and treatment responses.

Specimen No.	Diagnosis	Age and Sex	Initial stage	Specimen site	Genomic findings	Treatment and response prior dissection ( < 2 years)	Treatment and response post dissection ( < 2 years)
1	Pancreatic adenocarcinoma	55/Male	IV	Secondary–peritoneum	*BRCA2, KRAS, MTAP, CDKN2A/B*	–	1. Short term partial remission with Folinic acid, 5-FU, Irinotecan and Oxaliplatin. 2. Stable disease with Pembrolizumab and Lenvatinib.
2	Desmoplastic small round cell tumor of the peritoneum	27/Male	IV	Secondary–peritoneum	No alterations detected	–	1. Stable disease with Vincristine, Doxorubicin, Cyclophosphamide, Ifosfamide and Etoposide. 2. Stable disease with Irinotecan and temozolomide. 3. Deterioration with Pemmbrolizumab. 4. Stable disease with Cyclophosphamide and Topotecan.
3	Primary peritoneal carcinoma	67/Female	IV	Primary	*TP53, PIK3CA, MET, BRCA2*, uncertain significance, ER + , PR+	1. Partial remission with Paclitaxel and Carboplatin. 2. Short term stable disease with Bevacizumab and Doxil. 3. No response with Olaparib.	Deterioration with Topotecan Deceased
4	Moderately differentiated adenocarcinoma of large intestine	70/Male	IV	Secondary– liver	*TP53, KRAS, PIK3K, SMAD4*	Partial response with Folinic acid, 5-FU and Oxaliplatin.	– Deceased (not cancer related)
5	Pancreatic adenocarcinoma	71/Male	III	Primary	*TP53, KRAS, PIK3K, SMAD4*	–	– Deceased
6	Mucinous carcinoma of appendix	65/Female	IV	Secondary– peritoneum	*ATM*	–	1. Stable disease with Oxaliplatin and 5-FU. 2. Stable disease with Bevacizumab and 5-FU. Deceased
7	Squamous cell carcinoma of the anal canal	70/Male	IV	Secondary– liver	*KRAS, SMAD4, PIK3CA, MYC, BRCA1, EZH2, FABP5, PARP1, TOPOIIa, BIRC5*	1. Partial remission with Cisplatin and 5-FU. 2. Stable disease with Nivolumab. 3. No response with Paclitaxel.	1. Partial remission with Paclitaxel. 2. Deterioration with Mitomycin.
8	Pancreatic adenocarcinoma	44/Male	III	Primary	*TP53, BRCA2, KRAS, CDKN2A/B*	Stable disease with Folinic acid, 5-FU, Irinotecan and Oxaliplatin.	Partial response with Gemcitabine and Cisplatin.
9	Pancreatic neuroendocrine adenocarcinoma	56/Male	III	Primary	–	–	Somatostatin Cancer free
10	Adenocarcinoma of the colon	53/Female	IV	Secondary–peritoneum	*TP53, HER2*+*, APC*	–	1. Remission with Oxaliplatin, Capecitabine and Herceptin. 2. Remission with Herceptin.

Table detailing the diagnosis and disease course of ten cancer patients whose tumor samples were used to create personalized spheroids for chemotherapy testing.

**Fig. 5:**
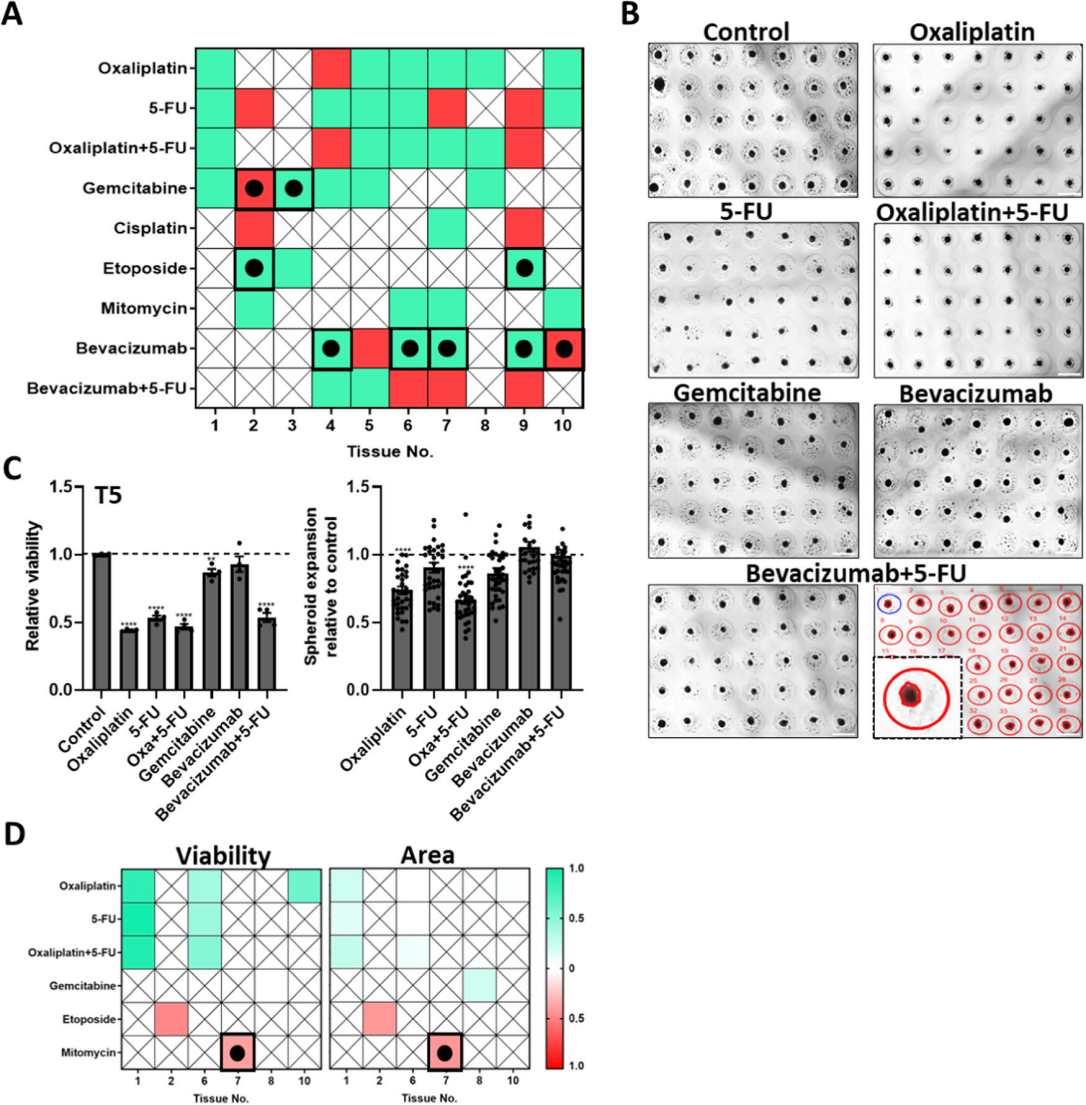
Chemotherapy testing on patient-derived tumor spheroids. **A** Color-coded map depicting the relationship between reduction in viability and spheroid area induced by drug treatment. A positive trend is indicated in the green boxes: both viability and spheroid area are reduced. The outlined green boxes marked with a dot indicate that both viability and area are enhanced. A reverse trend is indicated with red boxes: reduced viability with increased spheroid area. The outlined red boxes marked with a dot indicate enhanced viability with reduced spheroid area. **B** Representative images of spheroids formed from tumor cells extracted from a patient diagnosed with pancreatic adenocarcinoma (T5). The markings of the semi-automatic segmentation technique used to calculate the spheroid area are displayed on the bottom right. Scale bar = 1 mm. **C** Viability assay was performed on patient-derived spheroids 7 days after treatment initiation. *n* = 4. Area analysis is depicted as the area expansion on day 7 relative to the area 24 h after seeding. Data shown were normalized to control conditions (no added treatment: set to 1, shown as a dotted line). **D** Heat map representing the viability and area change of patient-derived spheroids 7 days after the addition of different treatments in correlation with clinical treatment outcomes. Green indicates on a direct trend between ex vivo experiment outcomes and clinical treatment outcomes (reduction in either viability or spheroid area and clinical responsiveness), while red indicates on an opposite trend (viability or spheroid area increase while clinical improvement is observed). The framed dotted red boxes indicate on a reverse trend with reduced viability or spheroid area while clinically there is deterioration. White indicates no significant change in either viability or area while clinically there is improvement with treatment. *n* = 48. ****p < 0.0001 and **p< 0.01 compared with control spheroids. Results are presented as mean ± SEM.

A common method used to quantify spheroid response to treatments is via sample size analysis^48–50^. To study whether spheroid size corresponded to its viability over time, the size and viability were monitored via image analysis of brightfield top-view microscopical images and the metabolism of tetrazolium salt respectively. The semi-automatic segmentation technique used to mark spheroid area is detailed in Fig. S7. A color-coded map depicting the relationship between reduction in viability and reduction in spheroid area induced by drug treatment is shown in Fig. 5a. A positive correlation between the changes in viability and spheroid area is observed in spheroids treated with Oxaliplatin, Gemcitabine, Etoposide or Mitomycin, as clearly observed in T5 spheroids (Fig. 5b, c). However, with 5-FU, Cisplatin or Bevacizumab, the correlation was frequently reversed. Similarly, RSL3, a ferroptosis inducer, caused a significant reduction in the viability of BeWo spheroids, while significantly increasing the spheroid area (Fig. S8). Figure 5d displays the correlation between patient-derived spheroid viability and area change with clinical treatment outcomes. T1, T6 and T10 spheroids display a direct trend in which enhanced reduction in viability correlates with clinical responsiveness. However, the spheroid area change is less prominent. The viability and area change of the other patient-derived spheroids are shown in Fig. S6. Fig. S9 displays BxPC-3 spheroid viability and area change over time after treatment with Cisplatin at various concentrations under flow conditions and static conditions. While the viability of spheroids is reduced in both flow and static conditions with increasing concentrations of Cisplatin, there is no significant change in the spheroid area under static conditions, whereas in flow conditions there is a significant increase in area ( ~ 3-fold) with the highest Cisplatin concentration.

## Discussion

Given the poor success rates of experimental drugs in clinical trials, fully humanized ex vivo models are becoming the preferred approach for disease modeling and drug development. Microfluidic chip technology is increasingly recognized as the next revolution in drug development and personalized medicine^51–54^. Despite the clear advantages of organ-on-a-chip technology, its widespread implementation is currently replete with drawbacks, such as flawed chip materials and long, laborious fabrication processes. Therefore, the goal of this study was to develop a novel technology that would provide a cost-effective solution for personalized drug treatment in cancer by enabling a more precise prediction of drug efficacy in tissue-like physiological conditions. For this purpose, we investigated suitable chip materials, developed protocols for enhancing material biocompatibility and conducted feasibility studies in clinical setups to compare ex vivo/in-patient correlations. This resulted in the generation of a fully 3D-printed device that is transparent, biocompatible, versatile, and sample accessible.

Most resins used as inks in digital light processing (DLP) are not transparent enough for receiving high resolution microscopy images^43^. This is partly due to their material properties, but also due to the voxel resolution of the printing process. To overcome this, a protocol for enabling commercially available resins to be printed directly onto glass slides was developed. This protocol was used to culture monolayer cells and 3D-cell aggregates in wells printed onto glass slides, producing high resolution images (Fig. 1).

The most commonly used materials for organ-on-a-chip microfluidics are PDMS and Poly(methyl-methacrylate) (PMMA), which are both considered compatible for cell-culture experiments^34,35^. PDMS can be used alone to fabricate microfluidic devices^55^ or in combination with other materials such as PMMA. This was demonstrated in work by Khot et al., where a microfluidic device comprised of a layer of PDMS sandwiched between two layers of PMMA was developed to generate, culture and analyze the cytotoxic effects of 5-FU on colorectal cancer spheroids^56^. However, PDMS and PMMA devices are less suitable for drug-based studies, mostly because they adsorb small hydrophobic molecules onto their surface. Also, their fabrication process requires specialized labor, resulting in low fabrication throughput^36,37,57,58^. Therefore, utilizing SLA 3D printing for the development of microfluidic devices is a preferred alternative since it considerably reduces fabrication time and labor while enabling versatility and complexity in device designs. The main downside of this fabrication method is the cytotoxicity of the photocurable resins used for printing, which occurs mainly as a result of the leakage of uncured resin monomers, such as acrylates, methacrylates, or urethanes^59,60^. In Fig. 2 and S2 we demonstrate the ability to generate fully 3D-printed biocompatible devices for the purpose of maintaining spheroids for long periods of time. The devices’ biocompatibility was demonstrated under “extreme” conditions. In addition to testing spheroid viability, cell monolayers were seeded in wells with the same diameter as 96-well plates under static conditions, lacking waste disposal. After all of the above testing, it may be concluded that since the spheroids are cultured in agarose molds and are therefore not in direct contact with the device and are grown under flow conditions where there is waste disposal, then the 3D-printed devices are a fortiori biocompatible for culturing patient-derived samples.

Once tissue samples are inserted into microfluidic devices, facile access to them is crucial for in-depth analysis and dynamic analysis over time. Many have tried to tackle this issue using two main approaches: extracting the biological sample^61–63^ or disassembling the microfluidic device^64–67^. Numerous microfluidic devices have been constructed in such a way that their different layers are sealed together, forming a “black box” where it is difficult to reach and analyze the cultured samples. Hence, the researcher must either extract them, as in the device developed by Chen et al.^63^, where analysis of the tumor spheroids necessitated their lysis prior to collection from the device, or the device must be dismantled. Phan et al.^65^ developed an arrayed high-throughput tumor-on-a-chip platform incorporated into a 96-well plate for drug screening purposes. It was constructed by removing the bottom of a 96-well plate and replacing it with a PDMS layer bonded to a thin transparent polymer membrane. A plastic protective sheet on the opposite side was kept, allowing the extraction of cells at the end of an experiment for further analysis. Extraction was accomplished by removing the plastic cover and slicing the desired PDMS region, rendering this platform nonreusable. Overcoming this, Schuster et al. developed an automated, high-throughput microfluidic device used for drug screening on human-derived pancreatic tumor organoids. The device was comprised of a glass slide nested between screws secured to a stage. Above was a thin layer of PDMS containing chamber wells with an additional PDMS layer containing channels. The final layer consisted of polycarbonate meant to reversibly bond the layers with knobs screwed down, enabling reusage of the platform^66^. However, to access the biological sample for in-depth analysis, the whole system needed to be disassembled, which could only be done after the experiment was terminated.

In contrast, we fabricated the devices depicted in Figs. 3, 4 to meet the need for total accessibility to the biological samples without disrupting the course of the experiment or sacrificing reusability. We based the culture of spheroids in these devices on two main spheroid assembly techniques: the hanging drop method and the ultra-low attachment surface method^46^. While many studies used devices containing bolts or clamps to fasten their systems^31,56,61,68^, our device used the culturing wells themselves to function as “nuts and bolts”, allowing access to the samples multiple times for long-term tracking and guaranteeing optimal sealing even while the experiment was ongoing. Also, our platform remained reusable for subsequent experiments.

Another of the system’s unique features is its versatility. Since the biological sample size is different for each type of cancer or patient, the system was designed to be compatible with molds of various sizes, such that the appropriate mold size can be selected for each sample to culture the desired number of spheroids (Fig. 4a).

The diversity and plasticity within tumors, combined with patient-specific factors, results in different therapeutic outcomes in different patients receiving the same treatments, leading to the need for individually tailored treatments^69^. Hence, the development of reliable 3D tumor models comprised of the patient’s own cells is of great value. In this study, the correlation between ex vivo results and clinical outcomes was examined by comparing the clinical data with the responses of multicellular spheroids derived from ten tumor biopsies treated with different chemotherapy combinations (Fig. 5 and S6). Despite the small-scale study, positive correlations were found in the clinical follow-up information. For instance, a ~90% reduction in viability was observed with spheroids comprised of T1 cells treated with Oxaliplatin and 5-FU. A similar trend appeared with a short-term partial remission using Folinic acid, 5-FU, Irinotecan and Oxaliplatin combination treatments (Table 1). The significant response to Oxaliplatin was consistent with the BRCA2 mutation the patient carried, which generally leads to a high response rate to platinum-containing regimens^44^. Furthermore, similar response trends were detected in the T4 and T5 spheroids, where both patients carried the same genomic abnormalities. T1, T6 and T10 spheroids displayed a direct trend in which enhanced reduction in viability correlated with clinical responsiveness; however, the spheroid area reduction was less prominent (Fig. 5d).

One of the most common ways to quantify spheroid response to treatments is via sample size analysis^48–50^. The accepted theory is that spheroids shrink in response to treatment and increase in size when grown under optimal conditions. This was largely what was found in patient-derived spheroids treated with either Oxaliplatin, Gemcitabine, Etoposide or Mitomycin (Fig. 5a). However, with 5-FU and Cisplatin, the relationship between reduction in viability and spheroid area was frequently anti-correlative. Similar phenomena have been observed in several studies^70–72^. For example, in a study conducted by Baek et al., some cancer spheroids became larger with increasing concentrations of Doxorubicin while others were not affected^73^, indicating that there are factors aside from viability that can affect spheroid size. A demonstration of this can be seen with BeWo cancer spheroids treated with RSL3, a ferroptosis inducer (Fig. S8). Ferroptosis is a form of cell death driven by cellular metabolism and iron-dependent lipid peroxidation involved in various diseases, including cancer^74^. In this case, a significant reduction in viability was associated with a significant increase in spheroid size. This phenomenon can be explained by the involvement of E-cadherin – known to be essential for cell-cell interactions in spheroid assembly^75^ and for the development of compact spheroids from loose aggregates^76^ – in regulating ferroptosis via the Hippo pathway^77^. There are many ferroptosis-associated anticancer drugs, among which are Cisplatin, 5-FU, Oxaliplatin and Gemcitabine. Cell death in these cases is induced mainly via GSH (lung and colorectal cancers^78^), Lipocalin 2 (colorectal cancer^79^) or GPX4 (colorectal and pancreatic cancer^80,81^) pathways. This demonstrates that the characteristics of the tumor itself, such as an iron-rich environment or specific gene expression (e.g., P53 tumor suppression pathway^82^), determine the effect drugs have on the mode of cell death^83,84^. Interestingly, while BxPC-3 cancer spheroids grown in static conditions and treated with increasing concentrations of Cisplatin displayed a significant reduction in viability, their area did not significantly change over time (Fig. S9), whereas the area of spheroids grown under flow conditions significantly increased with the highest Cisplatin concentration treatment. This might be the result of the combined anti-cancer treatment with the shear stress generated in the microfluidic device further promoting spheroid disintegration. It would be interesting to further investigate the relationship between different types of tumors and their genomic expression and the type of cell death induced by different anticancer drugs and the resulting effect on size. Given our observations, it can be concluded that size-based analysis can provide additional information in functional drug screening on patient-derived tumor spheroids, but it should be analyzed together with other functional tests to provide accurate conclusions.

## Methods

### Statement

All experiments and data collection were conducted in accordance with relevant guidelines and regulations. Informed consent was obtained from all subjects. All ethical regulations relevant to human research participants were followed. Human patient-derived cells were extracted from fresh tumor tissues as approved by the Institutional Review Board (IRB)/Ethics (Helsinki) committee of the Hadassah Medical Center (#920051034 and 0628-14-HMO).

### Cell culture

Human glioblastoma U87-MG, lung H460 and H2286, placenta BeWo, and pancreatic cancer cell lines BxPC-3, PANC-1 and AsPC-1 were obtained from ATCC (VA, USA). Prostate PC3M-LN4 cells were received from Bielenberg’s lab, as previously described in Brill-Karniely’s work^85^. All cells were mycoplasma free (using an EZ-PCR Mycoplasma Test Kit (Biological Industries) and used for the experiments up to passage 20). All cells were maintained in a 10% fetal calf serum (FCS) medium containing penicillin/streptomycin (P/S) and kept in a humidified incubator at 37 °C with 5% CO_2_ unless otherwise specified. For the U87-MG cell line, EMEM (Biological Industries, Israel) supplemented with 1% sodium pyruvate (Life Technologies, MA, USA) and 1% glutamine (Life Technologies) was used for cell culture. PC3M-LN4, H460, H2286, BxPC-3 and AsPC-1 cells were maintained in RPMI-1640 (Life Technologies). PANC-1 cells were grown in DMEM (Life Technologies). BeWo cells were grown in F-12K medium (ATCC) supplemented with 0.1% FCS. Patient-derived cells were isolated from cancer tissues by sectioning the tissue into ~1 mm pieces and digesting for 60–90 min at 37 °C with 5% CO_2_ while slowly stirring with a magnet in DMEM/F12 medium (Life Technologies) supplemented with P/S and 0.14 Wunsch units/ml of Liberase™ Research Grade (Roche Diagnostics). Digested tissue was filtered through a cell strainer, after which supernatant was diluted with a stop reaction medium (DMEM/F12 supplemented with P/S and 20% FCS) and centrifuged at 1,200 RPM for 5 min. Cells were plated and maintained in RPMI:F12:DMEM medium (1:1:3) supplemented with 10% FCS, 1% glutamine, P/S, HEPES (Biological Industries), 1% hydrocortisone (Sigma-Aldrich, MO, USA) and 5 ng/ml epithelial growth factor (EGF; ProSpec, Israel).

### 3D printing

All 3D-printed molds and microfluidic chips were fabricated using the digital light-processing stereolithography printer Asiga Max (Sydney, Australia) with a LED light source of 385 nm UV. All devices were designed in Autodesk AutoCad® (San Rafael, CA, USA). The final designs were exported as a stereolithography (STL) file and uploaded to the printer’s Asiga composer software for 3D printing (Sydney, Australia). The initial molds were printed with BV-007A resin (MIICRAFT, Jena, Germany), luxaprint® mould (Detax GmbH, Baden-Württemberg, Germany) and FREEPRINT® ortho (Detax GmbH). The devices and microfluidic chips intended for cell culture were all printed from FREEPRINT® ortho unless otherwise mentioned. The printed object was removed from the printing platform at the end of the printing process and uncured resin was removed by washing the print with ethanol for 5 min in a bath sonicator. The print was then cured under UV for 5 min (PCU Led, Dreve, Germany).

### Glass activation and 3D printing onto glass for cell culturing

Prior to the printing process, the glass slides 76x52x1 mm (MARIENFELD, München, Germany) were immersed in 2% 3-(Trimethoxysilyl)propyl methacrylate (TMSPMA) (Sigma-Aldrich) and then placed in an oven set to 105 °C. Afterward, the slides were mounted onto the printer’s platform to provide a new base onto which the object was printed. When the printing process was complete, the glass slide together with the printed object were removed and rinsed in 100% ethanol and then transferred to clean 100% ethanol and sonicated for 10 min. Following this, the prints were placed in a UV oven and cured for 1 h. Next, they were rinsed with DW four times and sterilized with UV for 30 min in a biological hood. Prior to seeding the cells, the glass bottoms were coated with Poly-L-Lysine (Sigma-Aldrich) for 1 h and rinsed four times with PBS.

### PDMS-based fabrication using 3D templates

PDMS (SYLGARD®184 silicone elastomer, MI, USA) was cast into 3D-printed templates containing complementary wells and placed in an oven set at 65 °C overnight. After curing, the PDMS cast was removed from the template and sterilized by exposure to UV light for 30 min in a biological hood.

### Seeding cells and spheroids in 3D-molds to examine mold transparency

BxPC-3 cells were seeded at 50,000 cells/well in standard polystyrene 48-well plates and molds 3D-printed onto glass slides containing wells with a diameter similar to a 48-well plate (radius = 5.91 mm). The cells were stained with Hoechst diluted 1:2000 with medium (Thermo Fischer Scientific, MA, USA) and imaged 24 h after seeding. Unless otherwise stated, all images were taken using a Nikon Eclipse Ti microscope (Tokyo, Japan).

Master 3D Petri Dish^®^ 35-well arrays (Microtissues Inc., RI, USA) were used to create microwells comprised of 2% UltraPure^TM^ Agarose hydrogel (Thermo Fischer Scientific). The microwells were seeded with BxPC-3 at 10,000 cells/well and after 24 h they were transferred into the wells of a FREEPRINT® ortho mold, PDMS mold, standard low-attachment surface 12-well plate or FREEPRINT® ortho mold printed onto a glass slide. Cell seeding density and duration of culture for receiving compact spheroids was based on our previous work^86^. Light microscopy images were taken with the Nikon Eclipse TS100 light microscope 48 h after transfer.

### Biocompatibility testing of 3D-printed molds

For the viability and proliferation assays, U87-MG, PC3M-LN4, BxPC-3 and H460 cells were seeded in standard 96-well plates and 3D-printed molds that were coated with PLL and that had the same diameter as the wells in the 96-well plates (radius = 3.19 mm) for 24 h and 96 h incubation (10,000 cells/well and 3000 cells/well respectively). This was followed by viability detection using WST1 reagent (Sigma-Aldrich, MO, USA). Absorbance was detected at 450 nm using a plate-reader (Wallac 1420 VICTOR plate-reader, Perkin-Elmer Life Sciences, Shelton, CT, USA).

96 h after seeding U87-MG, PC3M-LN4, BxPC-3 and H460 at 100,000 cells/well in standard 12-well plates and 3D-printed molds with the same well diameter as the wells in the 12-well plates (radius = 17.7 mm), the samples were stained using the Dead Cell Apoptosis kit with Annexin-V-APC and 7-Aminoactinomycin D (7-AAD) according to the manufacturer’s instructions (BioLegend, CA, USA). Samples were acquired using the LSRFortessa Flow Cytometer (BD Biosciences, NJ, USA) and analyses were conducted using FlowJo software (Tree Star, Ashland, OR, USA).

U87-MG, PC3M-LN4, BxPC-3 and H460 cells cultured under the same conditions as above mentioned were stained with Hoechst and Calcein AM and imaged (as previously mentioned), 96 h after their seeding.

U87-MG, BxPC-3 and H460 5000 cells/well were seeded in agarose microwells using the Master 3D Petri Dish^®^ 35-well arrays as previously mentioned. After 24 h the spheroids were transferred to standard 96-well plates and 3D-printed molds containing wells with similar radiuses as the wells in the 96-well plates (3.19 mm radius). After 96 h, all spheroids were stained with Calcein AM and imaged. Viability was detected using the WST1 reagent.

To compare the biocompatibility of different resins used for printing without using the optimal cleaning protocol, 3D-printed molds containing wells with the same dimensions as 96-well plates (radius = 0.319 mm) were printed straight onto glass slides using either Freeprint (ResinF), BV-007A (ResinB) or Luxaprint (ResinL). Each resin was printed twice, testing 2 different protocols of post-printing cleaning. They were either cleaned with ethanol or isopropanol and left in DDW overnight. PLL was used to coat the well bottoms and H2286, PC3M-LN4, BxPC-3 and U87-MG 5000 cells/well were seeded in the printed molds and in standard 96-well plates. WST1 reagent and cell staining with Hoechst and Calcein AM was conducted, as previously mentioned, 72 h after their seeding.

### Spheroid formation using hanging drop 3D-printed mold

U87-MG, PC3M-LN4, BxPC-3 cells were trypsinized and then stained with CellTrace^TM^ CFSE 1 uL/1 ml (Thermo Fischer Scientific, MA, USA) according to the manufacturer’s instructions. Each drop contained a total volume of 60 uL with 5,000 cells/drop. The cells were imaged 24 h and 48 h after their seeding into drops and transferred to 96-well plates coated with 2% agarose and imaged again after 24 h.

### Spheroid formation using ultra-low attachment microwells

3D-printed complementary templates were designed to contain either 7, 21 or 25 microwells. UltraPure^TM^ Agarose hydrogel 4% was cast inside, and when removed, microwells were formed allowing the seeding of cells inside. U87-MG, BxPC-3 and PC3M-LN4 were stained with Cell Proliferation Staining Reagent Green Fluorescence Cytopainter (ab176735, Abcam, Cambridge, UK) and immediately after, seeded 4000 cells/microwell. Images were taken 24 h after their seeding.

### 3D-printed “nut and bolt” chip for spheroid culture

The 3D-printed mixing gradient device and microfluidic chip for spheroid culture were made to flow with blue and yellow food coloring showing the color gradient formed. Next, blue food coloring and DDW were introduced into the mixing gradient device and the absorbance of the liquid collected at the outlets was measured at 570 nm using the Wallac 1420 VICTOR plate-reader.

### Computational Fluid Dynamics (CFD) simulation

Velocity flow simulations were conducted using the simulation software Autodesk CFD (2023). All wall boundaries in the printed devices were assumed to be smooth with a no-slip boundary condition. Inlet volume flow was set at 1 cm^3^/min or 10 cm^3^/min, while outlet pressures were set at atmospheric pressure (100 kPa). Simulations were implemented assuming an isothermal flow and Newtonian fluid behavior, including constant viscosity and density. Since the concentrations of the dissolved substances in the media were low, the properties of water at 37 °C were chosen for the simulations.

### Patient-derived cancer spheroids

Patient-derived cancer spheroids were formed using Master 3D Petri Dish^®^ 35-well arrays (as previously mentioned) from the following samples: pancreatic adenocarcinoma (T1, T5, T8), desmoplastic small round cell tumor of the peritoneum (T2), primary peritoneal carcinoma (T3), moderately differentiated adenocarcinoma of large intestine (T4), mucinous carcinoma of appendix (T6), squamous cell carcinoma of the anal canal (T7), pancreatic neuroendocrine carcinoma (T9) and adenocarcinoma of the colon (T10). All patient-derived spheroids were seeded at the same cell density (5000 cells/microwell); however, their initial sizes were slightly different. After 24 h of seeding, they were imaged and chemotherapy treatments were added. The chemotherapy drugs used were: Oxaliplatin, Gemcitabine, Etoposide, Mitomycin, 5-FU, Cisplatin and Bevacizumab. The last one provided a negative control since its anticancer activity is mainly accomplished via inhibition of angiogenesis. The spheroids were once again imaged 7 d after treatment addition. WST1 reagent was used to measure viability as previously mentioned.

### Statistics and reproducibility

Statistical data was analyzed on GraphPad Prism 9 (www.graphpad.com, San Diego CA) and all experiments had at least three independent replicates. Studies containing two groups were assessed using the unpaired two-tailed Student’s *t*-test. Studies containing more than three groups were compared and analyzed using a one-way analysis of variance (ANOVA), and significant differences were detected using Tuckey’s multiple comparison post-test. Differences were considered statistically significant for *p* < 0.05.

### Supplementary information

Supplementary information

Description of Additional Supplementary Files

Supplementary Data 1

Movie S1

## Data Availability

Source data for the graphs in the main figures is available as Supplementary Data 1, and any remaining information can be obtained from the corresponding author upon reasonable request.

## Code availability

Code can be made available upon reasonable request from the authors.

## References

[1] Grzywa TM, Paskal W, Włodarski PK (2017). Intratumor and intertumor heterogeneity in melanoma. Transl. Oncol..

[2] Burrell RA, McGranahan N, Bartek J, Swanton C (2013). The causes and consequences of genetic heterogeneity in cancer evolution. Nature.

[3] Ahn DH, Ciombor KK, Mikhail S, Bekaii-Saab T (2016). Genomic diversity of colorectal cancer: changing landscape and emerging targets. World J. Gastroenterol..

[4] Niu B (2016). Protein-structure-guided discovery of functional mutations across 19 cancer types. Nat. Genet..

[5] Xu X, Farach-Carson MC, Jia X (2014). Three-dimensional in vitro tumor models for cancer research and drug evaluation. Biotechnol. Adv..

[6] Lamichhane SP (2016). Recapitulating epithelial tumor microenvironment in vitro using three dimensional tri-culture of human epithelial, endothelial, and mesenchymal cells. BMC Cancer.

[7] Klimkiewicz K (2017). A 3D model of tumour angiogenic microenvironment to monitor hypoxia effects on cell interactions and cancer stem cell selection. Cancer Lett..

[8] Cavo M (2016). Microenvironment complexity and matrix stiffness regulate breast cancer cell activity in a 3D in vitro model. Sci. Rep..

[9] Shannon AM, Bouchier-Hayes DJ, Condron CM, Toomey D (2003). Tumour hypoxia, chemotherapeutic resistance and hypoxia-related therapies. Cancer Treat. Rev..

[10] Pampaloni F, Reynaud EG, Stelzer EHK (2007). The third dimension bridges the gap between cell culture and live tissue. Nat. Rev. Mol. Cell Biol..

[11] Hirschhaeuser F (2010). Multicellular tumor spheroids: an underestimated tool is catching up again. J. Biotechnol..

[12] Fontoura JC (2020). Comparison of 2D and 3D cell culture models for cell growth, gene expression and drug resistance. Mater. Sci. Eng. C.

[13] Jensen C, Teng Y (2020). Is it time to start transitioning from 2D to 3D cell culture?. Front. Mol. Biosci..

[14] Duval K (2017). Modeling physiological events in 2D vs. 3D cell culture. Physiology.

[15] Pinto B, Henriques AC, Silva PMA, Bousbaa H (2020). Three-dimensional spheroids as in vitro preclinical models for cancer research. Pharmaceutics.

[16] Gilazieva Z, Ponomarev A, Rutland C, Rizvanov A, Solovyeva V (2020). Promising applications of tumor spheroids and organoids for personalized medicine. Cancers.

[17] Zanoni M (2016). 3D tumor spheroid models for in vitro therapeutic screening: a systematic approach to enhance the biological relevance of data obtained. Sci. Rep..

[18] Sant S, Johnston PA (2017). The production of 3D tumor spheroids for cancer drug discovery. Drug Discovery Today Technol..

[19] Groebe K, Mueller-Klieser W (1991). Distributions of oxygen, nutrient, and metabolic waste concentrations in multicellular spheroids and their dependence on spheroid parameters. Eur. Biophys. J..

[20] Harris AL (2002). Hypoxia - A key regulatory factor in tumour growth. Nat. Rev. Cancer.

[21] Semenza GL (2003). Targeting HIF-1 for cancer therapy. Nat. Rev. Cancer.

[22] Phelps AS- (2019). Modelling cancer in microfluidic. Nat. Rev. Cancer.

[23] Pasini A (2021). Perfusion flow enhances viability and migratory phenotype in 3D-cultured breast cancer cells. Ann. Biomed. Eng..

[24] Azimi T, Loizidou M, Dwek MV (2020). Cancer cells grown in 3D under fluid flow exhibit an aggressive phenotype and reduced responsiveness to the anti-cancer treatment doxorubicin. Sci Rep.

[25] Huang Q (2018). Fluid shear stress and tumor metastasis. Am. J. Cancer Res..

[26] Sleeboom JJF, Amirabadi HE, Nair P, Sahlgren CM, Den Toonder JMJ (2018). Metastasis in context: Modeling the tumor microenvironment with cancer-on-a-chip approaches. DMM Dis. Models Mech..

[27] Bracken MB (2009). Why animal studies are often poor predictors of human reactions to exposure. J. R. Soc. Med..

[28] Van Norman GA (2020). Limitations of animal studies for predicting toxicity in clinical trials: Part 2: potential alternatives to the use of animals in preclinical trials. JACC Basic to Transl. Sci..

[29] Martić-Kehl MI, Schibli R, Schubiger PA (2012). Can animal data predict human outcome? Problems and pitfalls of translational animal research. Eur. J. Nucl. Med. Mol. Imaging.

[30] Wadman M (2023). FDA no longer needs to require animal tests before human drug trials. Science.

[31] Liu X (2021). Tumor-on-a-chip: from bioinspired design to biomedical application. Microsyst. Nanoeng..

[32] Bērziņa S, Harrison A, Taly V, Xiao W (2021). Technological advances in tumor-on-chip technology: from bench to bedside. Cancers.

[33] Imparato G, Urciuolo F, Netti PA (2022). Organ on chip technology to model cancer growth and metastasis. Bioeng.

[34] Au AK, Lee W, Folch A (2014). Mail-order microfluidics: evaluation of stereolithography for the production of microfluidic devices. Lab Chip.

[35] Sackmann, E. K., Fulton, A. L. & Beebe, D. J. The present and future role of microfluidics in biomedical research. *Nature* **507**, 181–189 (2014). 10.1038/nature13118 24622198

[36] Auner AW, Tasneem KM, Markov DA, McCawley LJ, Hutson MS (2019). Chemical-PDMS binding kinetics and implications for bioavailability in microfluidic devices. Lab Chip.

[37] Kakuta M, Bessoth FG, Manz A (2001). Microfabricated devices for fluid mixing and their application for chemical synthesis. Chem. Rec..

[38] Toepke MW, Beebe DJ (2006). PDMS absorption of small molecules and consequences in microfluidic applications. Lab Chip.

[39] Rodriguez AD (2020). A microfluidic platform for functional testing of cancer drugs on intact tumor slices. Lab Chip.

[40] Lee J, Kim M (2022). Polymeric microfluidic devices fabricated using epoxy resin for chemically demanding and day-long experiments. Biosensors.

[41] Pagac M (2021). A review of vat photopolymerization technology: materials, applications, challenges, and future trends of 3D printing. Polym.

[42] Bhattacharjee N, Urrios A, Kang S, Folch A (2016). The upcoming 3D-printing revolution in microfluidics. Lab Chip.

[43] Urrios A (2016). 3D-printing of transparent bio-microfluidic devices in PEG-DA. Lab Chip.

[44] Lee A, Moon BI, Kim TH (2020). BRCA1/BRCA2 Pathogenic variant breast cancer: treatment and prevention strategies. Ann. Lab. Med..

[45] Guerrero-Gironés J (2022). In vitro biocompatibility testing of 3D printing and conventional resins for occlusal devices. J. Dent..

[46] Timmins NE, Nielsen LK (2007). Generation of multicellular tumor spheroids by the hanging-drop method. Methods Mol. Med..

[47] Białkowska K, Komorowski P, Bryszewska M, Miłowska K (2020). Spheroids as a type of three-dimensional cell cultures—examples of methods of preparation and the most important application. Int. J. Mol. Sci..

[48] Shahi Thakuri, P., Gupta, M., Plaster, M. & Tavana, H. Quantitative size-based analysis of tumor spheroids and responses to therapeutics. *Drug Dev. Technol*. 10.1089/adt.2018.895 (2019). 10.1089/adt.2018.895 PMC6599382 30958703

[49] Aguilar Cosme, J. R., Gagui, D. C., Bryant, H. E. & Claeyssens, F. Morphological response in cancer spheroids for screening photodynamic therapy parameters. *Front. Mol. Biosci.*10.3389/fmolb.2021.784962 (2019). 10.3389/fmolb.2021.784962 PMC8637197 34869604

[50] Vinci M (2012). Advances in establishment and analysis of three-dimensional tumor spheroid-based functional assays for target validation and drug evaluation. BMC Biol..

[51] Pattanayak P (2021). Microfluidic chips: recent advances, critical strategies in design, applications and future perspectives. Microfluid. Nanofluid..

[52] Maurya R (2022). Advances in microfluidics devices and its applications in personalized medicines. Prog. Mol. Biol. Transl. Sci.

[53] Liu Y, Sun L, Zhang H, Shang L, Zhao Y (2021). Microfluidics for drug development: from synthesis to evaluation. Chem. Rev..

[54] Elvira KS (2021). Microfluidic technologies for drug discovery and development: friend or foe?. Trends Pharmacol. Sci..

[55] Torino S, Corrado B, Iodice M, Coppola G (2018). PDMS-based microfluidic devices for cell culture. Invent.

[56] Ibrahim Khot, M. et al. Characterising a PDMS based 3D cell culturing microfluidic platform for screening chemotherapeutic drug cytotoxic activity. *Sci. Rep.*10.1038/s41598-020-72952-1 (2020). 10.1038/s41598-020-72952-1 PMC7522244 32985610

[57] Van Midwoud PM, Janse A, Merema MT, Groothuis GMM, Verpoorte E (2012). Comparison of biocompatibility and adsorption properties of different plastics for advanced microfluidic cell and tissue culture models. Anal. Chem..

[58] Giri K, Tsao CW (2022). Recent advances in thermoplastic microfluidic bonding. Micromachines.

[59] Fritschen A (2022). Investigation and comparison of resin materials in transparent DLP-printing for application in cell culture and organs-on-a-chip. Biomater. Sci..

[60] Carve M, Wlodkowic D (2018). 3D-printed chips: compatibility of additive manufacturing photopolymeric substrata with biological applications. Micromachines.

[61] Petreus T (2021). Tumour-on-chip microfluidic platform for assessment of drug pharmacokinetics and treatment response. Commun. Biol.

[62] Saha B (2021). Human tumor microenvironment chip evaluates the consequences of platelet extravasation and combinatorial antitumor-antiplatelet therapy in ovarian cancer. Sci. Adv..

[63] Chen Y, Gao D, Liu H, Lin S, Jiang Y (2015). Drug cytotoxicity and signaling pathway analysis with three-dimensional tumor spheroids in a microwell-based microfluidic chip for drug screening. Anal. Chim. Acta..

[64] Riley A (2019). A novel microfluidic device capable of maintaining functional thyroid carcinoma specimens ex vivo provides a new drug screening platform. BMC Cancer.

[65] Phan DTT (2017). A vascularized and perfused organ-on-a-chip platform for large-scale drug screening applications. Lab Chip.

[66] Schuster B (2020). Automated microfluidic platform for dynamic and combinatorial drug screening of tumor organoids. Nat. Commun..

[67] Horowitz LF (2020). Multiplexed drug testing of tumor slices using a microfluidic platform. Npj Precis. Oncol..

[68] Skardal A (2017). Multi-tissue interactions in an integrated three-tissue organ-on-a-chip platform. Sci. Rep..

[69] Mathur L, Ballinger M, Utharala R, Merten CA (2020). Microfluidics as an enabling technology for personalized cancer therapy. Small.

[70] Maietta I (2022). Synergistic antitumoral effect of epigenetic inhibitors and gemcitabine in pancreatic cancer cells. Pharmaceuticals.

[71] Wade, S. J. et al. Dual delivery of gemcitabine and Paclitaxel by wet-spun coaxial fibers induces pancreatic ductal adenocarcinoma cell death, reduces tumor volume, and sensitizes cells to radiation. *Adv. Healthc. Mater* **9**, e2001115 (2020). 10.1002/adhm.202001115 33000905

[72] Zhao Y (2021). M1 Macrophage-derived exosomes loaded with gemcitabine and deferasirox against chemoresistant pancreatic cancer. Pharmaceutics.

[73] Baek N, Seo OW, Kim M, Hulme J, An SS (2016). Monitoring the effects of doxorubicin on 3D-spheroid tumor cells in real-time. Onco. Targets. Ther..

[74] Stockwell BR (2017). Ferroptosis: A regulated cell death nexus linking metabolism, redox biology, and disease. Cell.

[75] Shimazui T (2004). Role of complex cadherins in cell-cell adhesion evaluated by spheroid formation in renal cell carcinoma cell lines. Oncol. Rep..

[76] Lin Li-Fang Chou Chi-Chen Michael Chien Hwan-You Chang R-Z (2006). Dynamic analysis of hepatoma spheroid formation: roles of E-cadherin and β1-integrin. Cell Tissue Res..

[77] Dietrich C, Hofmann TG (2021). Ferroptosis meets cell–cell contacts. Cells.

[78] Roh JL, Kim EH, Jang HJ, Park JY, Shin D (2016). Induction of ferroptotic cell death for overcoming cisplatin resistance of head and neck cancer. Cancer Lett..

[79] Chaudhary N (2021). Lipocalin 2 expression promotes tumor progression and therapy resistance by inhibiting ferroptosis in colorectal cancer. Int. J. Cancer.

[80] Yang C, Zhang Y, Lin S, Liu Y, Li W (2021). Suppressing the KIF20A/NUAK1/Nrf2/GPX4 signaling pathway induces ferroptosis and enhances the sensitivity of colorectal cancer to oxaliplatin. Aging.

[81] Zhu S (2017). HSPA5 regulates ferroptotic cell death in cancer cells. Cancer Res..

[82] Jiang, L. et al. Ferroptosis as a p53-mediated activity during tumour suppression. *Nature*10.1038/nature14344 (2015). 10.1038/nature14344 PMC4455927 25799988

[83] Zhang C, Liu X, Jin S, Chen Y, Guo R (2022). Ferroptosis in cancer therapy: a novel approach to reversing drug resistance. Mol. Cancer.

[84] Zhao L (2022). Ferroptosis in cancer and cancer immunotherapy. Cancer Commun..

[85] Brill-Karniely Y (2020). Triangular correlation (TrC) between cancer aggressiveness, cell uptake capability, and cell deformability. Sci. Adv..

[86] Shoval H (2017). Tumor cells and their crosstalk with endothelial cells in 3D spheroids OPEN. Sci. Rep..

