# Supplementary material for: A fully 3D-printed versatile tumor-on-a-chip allows multi-drug screening and correlation with clinical outcomes for personalized medicine
Source: Commun Biol. 2023 Nov 13;6:1157. doi: 10.1038/s42003-023-05531-5 (PMC10643569; doi:10.1038/s42003-023-05531-5)
Supplement: Supplementary file 1 — Supplementary information [file 42003_2023_5531_MOESM1_ESM.pdf]

Supplementary Methods

IC_{50} calculation of chemotherapy drugs

Pancreatic cancer cells BxPC-3, PANC-1, and AsPC-1 were seeded 8,000 cells/well in 96-well plates. 24 h after seeding, chemotherapy treatments were added using 0, 1, 10, 50, 100 and 500 µM concentrations and left for 72 h followed by the WST1 assay conducted as previously mentioned. All chemotherapy drugs were obtained from the cytotoxicity unit at the Hadassah Medical Center.

Semi-automatic segmentation of cell aggregates

In order to make area measurements easier to calculate, semi-automatic segmentation techniques were used. These were then used as approximations of the true segmentations which were manually fixed. The semi-automatic segmentation was produced in two stages: finding wells in the mold and then segmenting the aggregates. Both of these stages utilized tools and implementations from Python's scikit-image (https://scikit-image.org/). The whole process is wrapped in a user interface implemented in Python's Bokeh (https://bokeh.org/) package in order to allow interactive refinement of the segmentations. Code can be found at: https://github.com/friedmanroy/CIDR-cell-segment.

In order to find the wells, circle detection was used through Hough transform on the image after processing with a Canny edge detector with $\sigma=0.3$, a low threshold of 0.3 and a high threshold of 0.7. After finding the wells, the image of each well was extracted from the full mold and processed according to the following steps:

1. The image was normalized by removing the minimum value and dividing by the maximum value.
2. A median filter with a kernel of size 5 was applied on the image to remove noise and small dust specks.
3. The histogram of the image was equalized with contrast-limited adaptive histogram equalization to make sure that the histogram was similar between different images.

In order to find the approximate segmentation, hysteresis thresholding was used with manually chosen quantiles, typically ~87% for the low threshold and ~95% for the high threshold. Binary closing was then used on the resulting image in order to remove small holes that might appear in the segmentation. Finally, the contour of the segmentation was found using scikit-image's "find contours" function, which was used to define a polygon that the user could edit in order to refine the segmentation. To calculate the area of the segmentation, the area of the polygon was calculated in pixels, which was then multiplied by the squared micron-to-pixel ratio in order to get the measurement in micron^2 units. The micron-to-pixel ratio was calculated using the well radius, which was fixed across different mold types.

Cancer spheroids treated with Cisplatin in a “nut and bolt” chip

BxPC-3 cells were seeded at 8,000 cells/spheroid in 7-microwell agarose molds prepared using the previously mentioned 3D-printed complementary templates. Molds containing the spheroids were placed in the “nut and bolt” microfluidic device or in a 96-well plate. Spheroids were treated with various concentrations of Cisplatin at a flow rate of 0.001 ml/min using a Chemyx F200X syringe pump (TX, USA) or grown under static conditions respectively 72 h after their seeding. WST1 reagent was used to test viability 5 d after treatment initiation, as previously mentioned. Images taken 72 h after seeding and 5 d after treatment initiation were used to analyze the change in spheroids’ area over time.

Induction of ferroptosis in cancer spheroids
BeWo cells were seeded at 5,000 cells/microwell in Master 3D Petri Dish® 35-well arrays as previously mentioned. The spheroids were imaged 48 h after seeding, and RSL3 (Sigma-Aldrich) 250 nM or RSL3 1,250 nM, and Ferrostatin-1 (Fer1) 0.5 µM (Sigma-Aldrich) were added for a 24 h incubation. After 24 h, the spheroids were imaged again, and viability and area measurement were conducted as previously mentioned.

Supplementary Figures

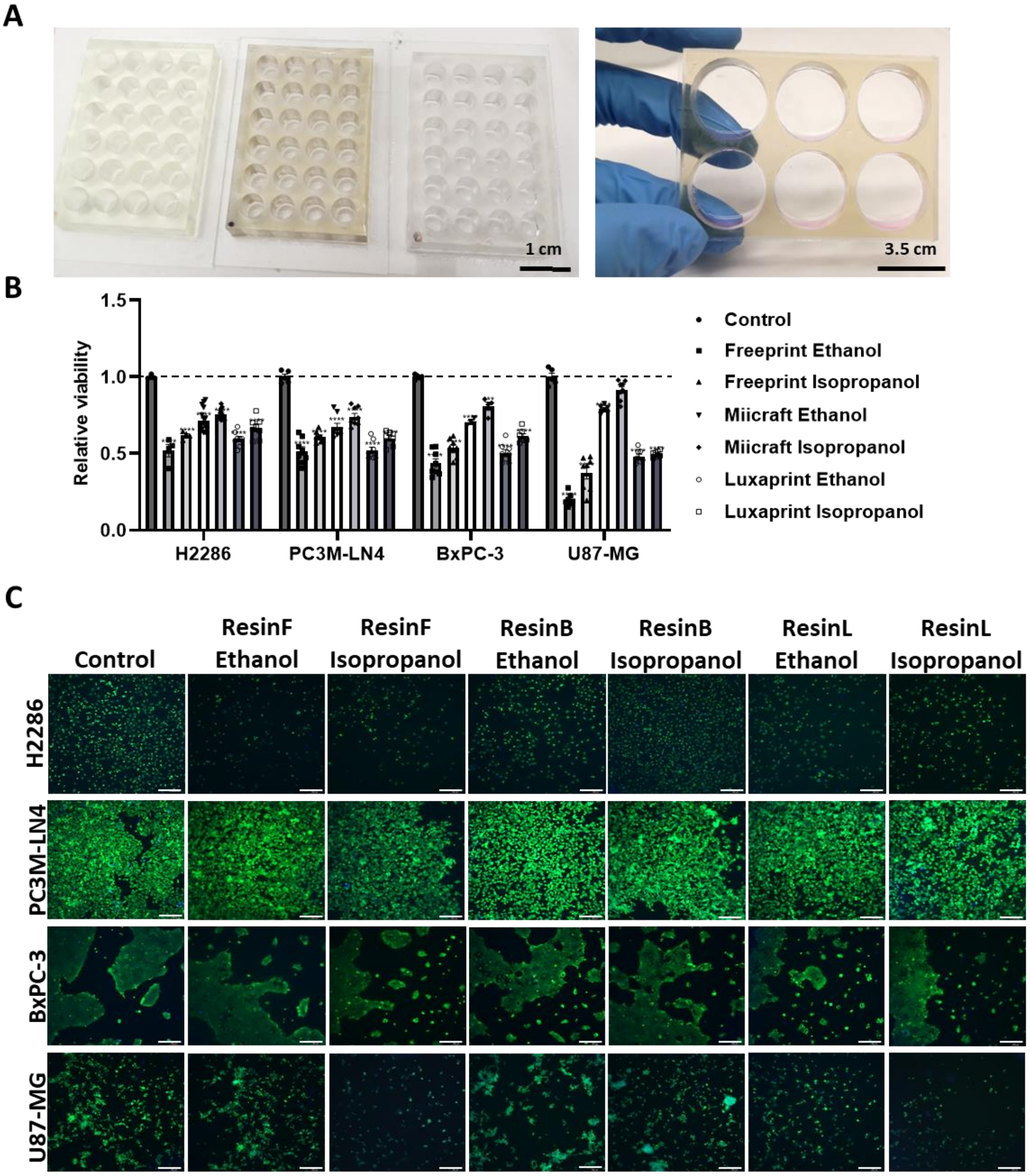

Fig. S1. Long-term culture of cells in 3D-printed molds indicates high cytotoxicity. (**A**) Image of molds printed on glass slides. Left - wells with 0.319 mm radius (similar to standard 96-well plates) and right - wells with 17.7 mm radius (similar to standard 12-well plates). 3D-printed molds containing wells with the same dimensions as 96-well plates (radius=0.319 mm) were printed straight onto glass

slides using either ResinF, ResinB or ResinL. Each resin was printed twice, testing 2 different protocols of post-printing processing. They were either cleaned with ethanol or isopropanol and left in DDW overnight. (**B**) PLL was used to coat the well bottoms and H2286, PC3M-LN4, BxPC-3 and U87-MG 5,000 cells/well were seeded in the printed molds and in standard 96-well plates (control). WST1 reagent was added 72 h after their seeding and absorbance was measured at 450 nm. (**C**) The cells were stained with Hoechst and Calcein AM 72 h after their seeding and imaged. Scale bar=200 um. n=6-8. ****p<0.0001 and ***p<0.001 compared with control cells. Results are presented as mean ± SEM.

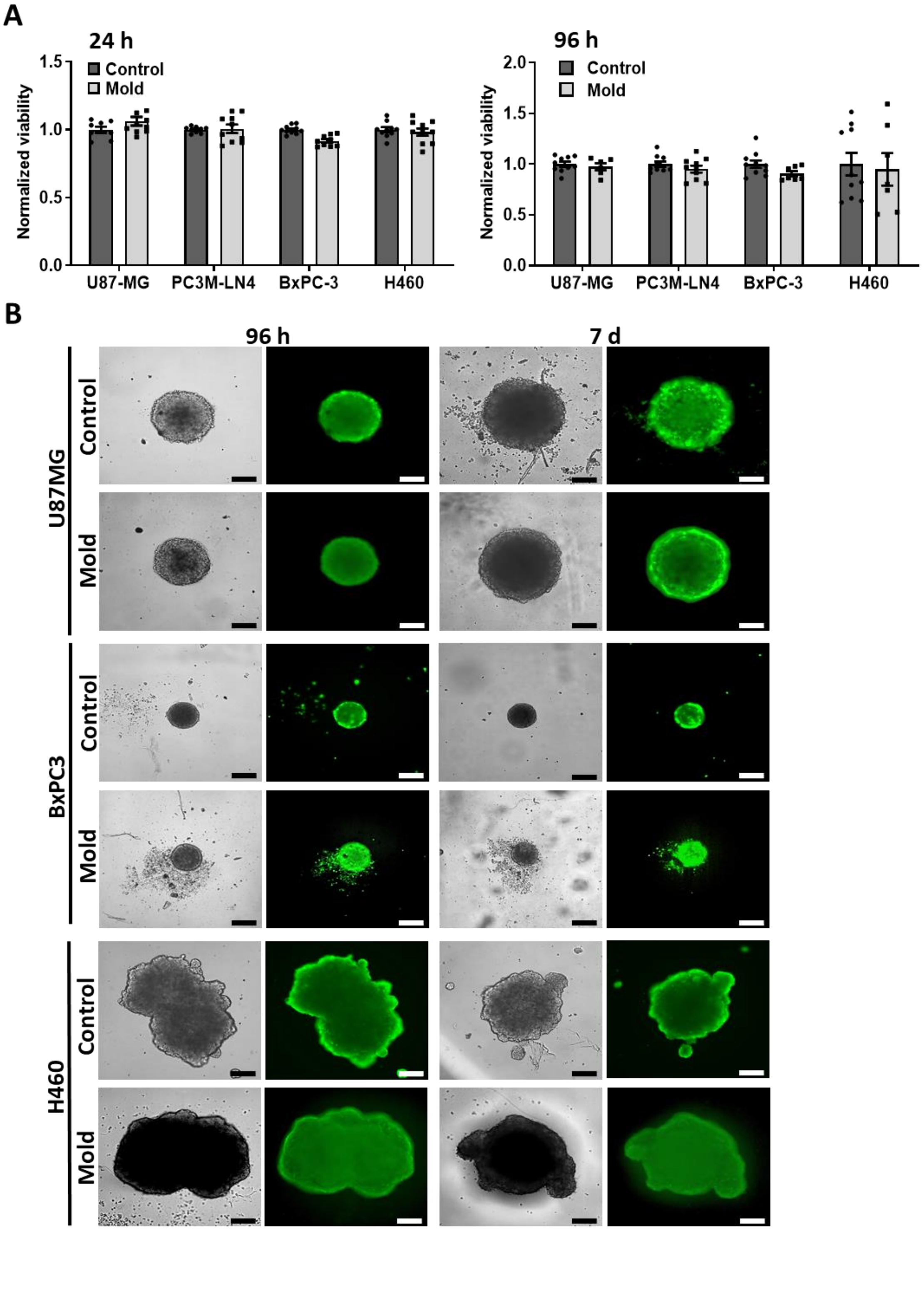

Fig. S2. Long-term culture of cells and spheroids in 3D-printed molds indicates its high biocompatibility. (**A**) U87-MG, PC3M-LN4, BxPC-3 and H460 cells were seeded in standard regular 96-well plates (control) and 3D-printed molds containing the same diameter as the wells in the 96-well plates (radius=3.19 mm) for 24 and 96 h incubation (10,000 cells/well and 3,000 cells/well respectively). WST1 was added into each well 24 and 96 h after seeding and absorbance was read at 450 nm using the Wallac 1420 VICTOR plate-reader. n=8. All results are statistically not significant. (**B**) U87-MG, BxPC-3 and H460 were seeded 5,000 cells/microwells and after 24 h were transferred to 3D-printed 3.19 mm radius wells or 96-well standard plates (control). After 96 h and 7 d the spheroids were stained with Calcein AM. Scale bar=200 um. n=3-4.

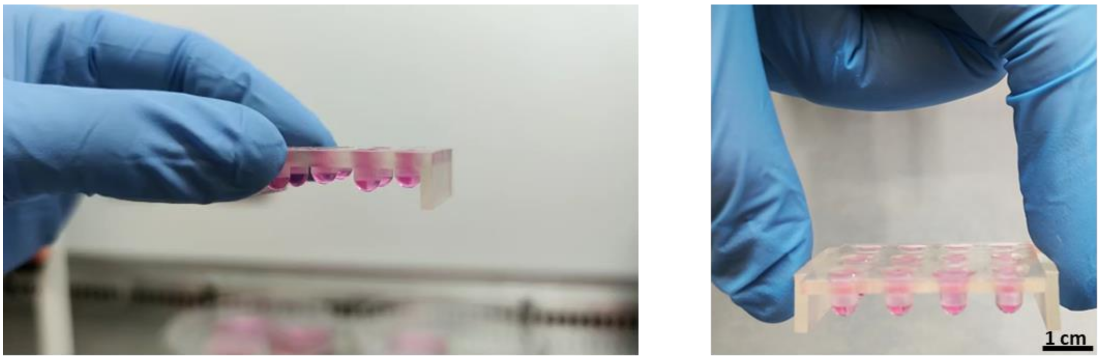

Fig. S3. Spheroid formation in 3D-printed mold utilizing the hanging drops method. Images of hanging drops in a printed mold containing the optimal geometry for stable drops 5 days after they were formed.

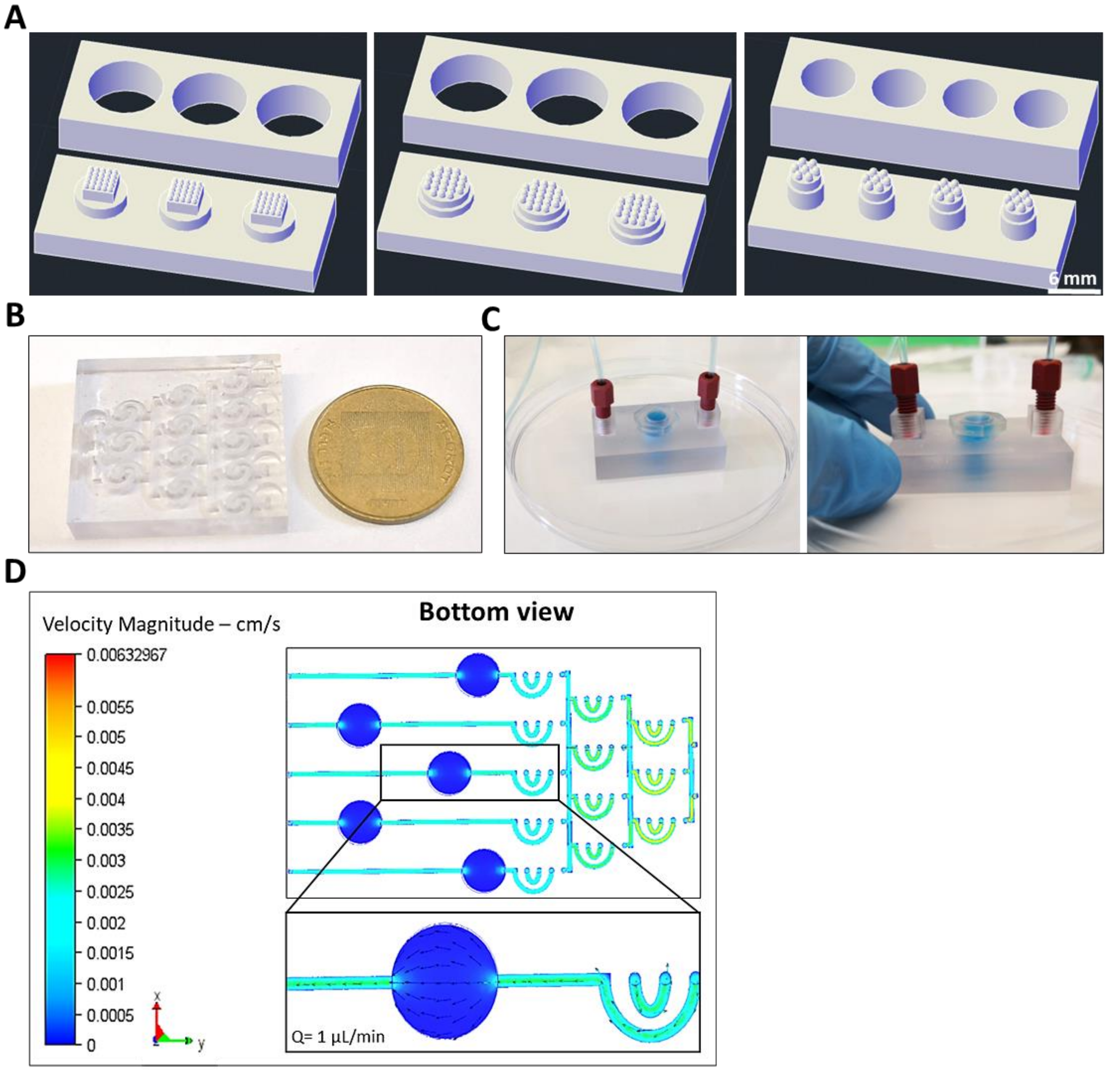

Fig. S4. 3D-printed "nut and bolt" chip for spheroid culture using the ultra-low attachment microwell method. (**A**) AutoCad® design of complementary templates used to form UltraPure^{TM} Agarose hydrogel microwells containing either 7, 21 or 25 wells. (**B**) Microfluidic device designed with spiral channels extending over several plains, enabling maximum mixing in minimal space. (**C**) Blue food coloring flowed through "nut and bolt" microfluidic device, demonstrating good sealing and no leakage. (**D**) Top view of a simulated flow velocity profile of the bottom layer of the full microfluidic device using a perfusion rate of 0.001 cm^3/min.

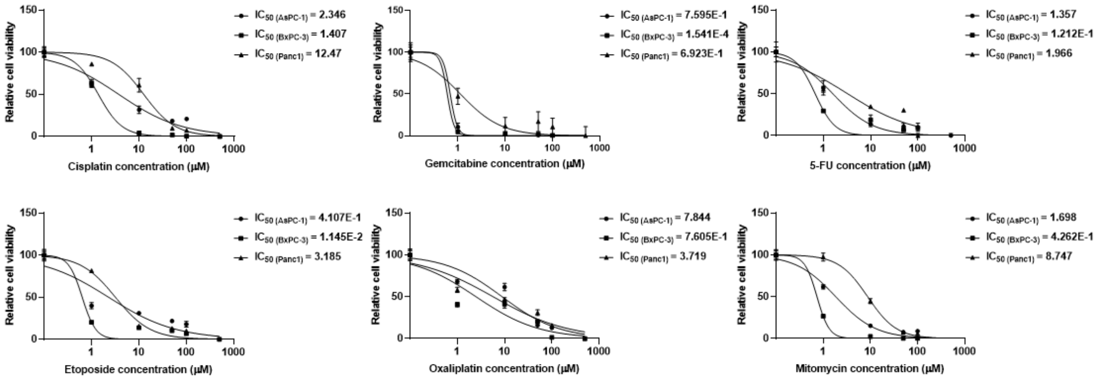

Drug	Cisplatin	Gemcitabine	5-FU	Etoposide	Oxaliplatin	Mitomycin
**AsPC-1 IC50 (µM)**	2.346	7.595E-1	1.357	4.107E-1	7.844	1.698
**BxPC-3 IC50 (µM)**	1.407	1.541E-4	1.212E-1	1.145E-2	7.605E-1	4.262E-1
**Panc1 IC50 (µM)**	12.47	6.923E-1	1.966	3.185	3.719	8.747

Fig. S5. IC50 calculation using pancreatic cancer cells. Pancreatic cancer cells BxPC-3, PANC-1, and AsPC-1 were seeded at 8,000 cells/well in 96-well plates. 24 h after seeding, chemotherapy treatments were added using 0, 1, 10, 50, 100 and 500 µM concentrations and followed with the addition of WST1 reagent after 72 h. The IC50s presented in the graphs and table are normalized so that the highest inhibition induced is considered as 100%.

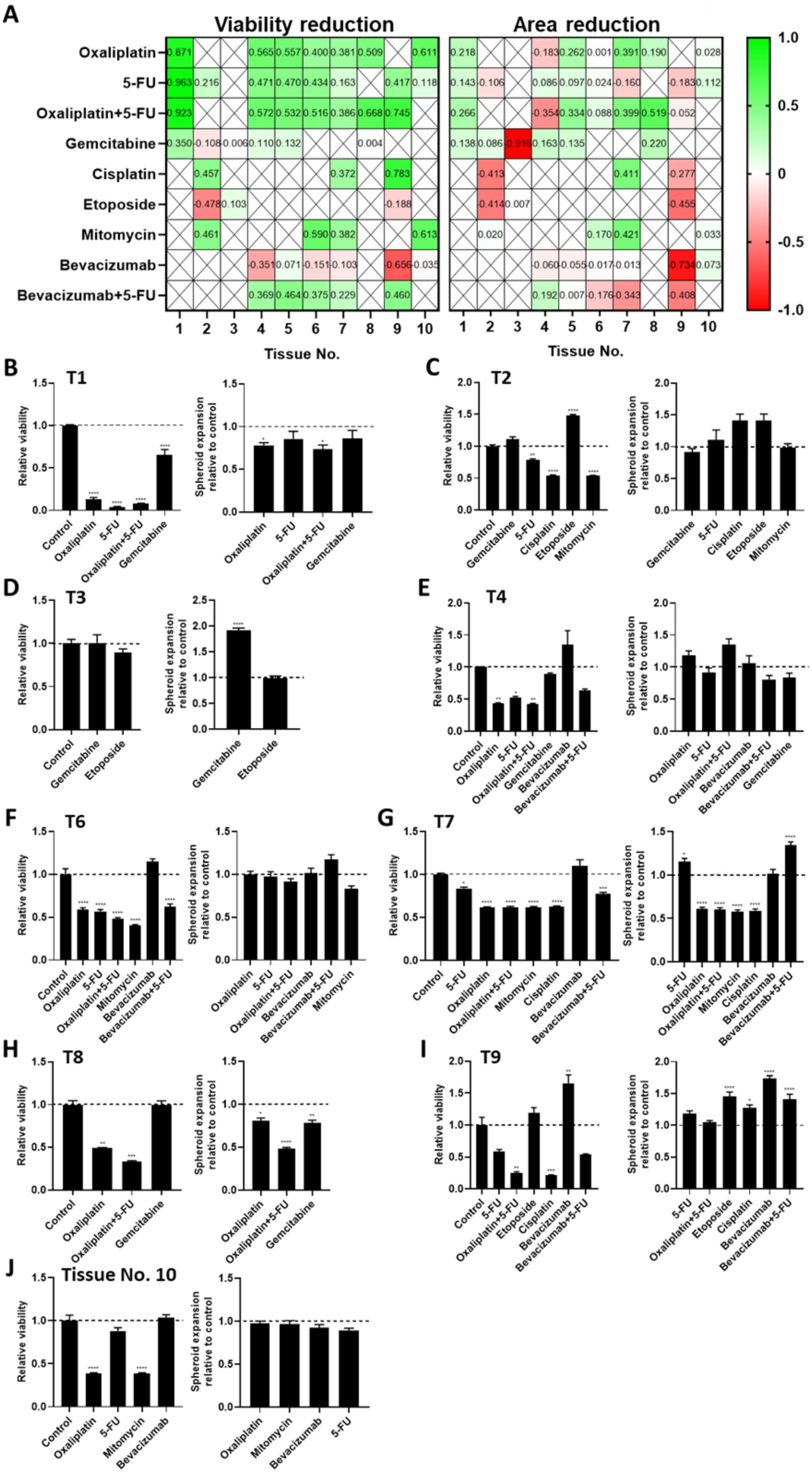
A
Viability reduction
Area reduction
Oxaliplatin
5-FU
Oxaliplatin+5-FU
Gemcitabine
Cisplatin
Etoposide
Mitomycin
Bevacizumab
Bevacizumab+5-FU
0.871 0.565 0.557 0.400 0.381 0.509 0.611
0.963 0.216 0.471 0.470 0.434 0.163 0.417 0.118
0.923 0.572 0.532 0.516 0.386 0.668 0.745
0.350 -0.108 0.006 0.110 0.132 0.004
0.457 0.372 0.783
-0.478 0.103 -0.188
0.461 0.590 0.382 0.613
-0.351 0.071 -0.151 -0.103 -0.656 -0.035
0.369 0.464 0.375 0.229 0.460
0.218 -0.183 0.262 0.001 0.391 0.190 0.028
0.143 -0.106 0.086 0.097 0.024 -0.160 -0.183 0.112
0.266 -0.354 0.334 0.088 0.399 0.519 -0.052
0.138 0.086 -0.916 0.163 0.135 0.220
-0.413 0.411 -0.277
-0.414 0.007 -0.455
0.020 0.170 0.421 0.033
-0.060 -0.055 -0.017 -0.013 -0.734 0.073
0.192 0.007 -0.176 -0.343 -0.408
1.0
0.5
0
-0.5
-1.0
Tissue No.
B T1
C T2
D T3
E T4
F T6
G T7
H T8
I T9
J Tissue No. 10
Relative viability
Spheroid expansion relative to control
Control
Oxaliplatin
5-FU
Oxaliplatin+5-FU
Gemcitabine
Cisplatin
Etoposide
Mitomycin
Bevacizumab
Bevacizumab+5-FU

Fig. S6. Viability and area analyses of patient-derived spheroids treated with chemotherapies. (**A**) Heat map representing the viability and area reduction of patient-derived spheroids 7 d after the addition of different treatments. A reduction in either viability or spheroid area is indicated in green, while an increase in viability or spheroid area is indicated in red. No significant change in either viability or area is indicated in white. A viability assay was performed on patient-derived spheroids 7 d after treatment initiation. n=4. Area analysis is depicted as the area expansion on day 7 relative to 24 h after seeding. Data shown were reanalyzed by relating the response to a given treatment to that under control conditions (no added treatment: set to 1, shown as a dotted line) for spheroids obtained from patients with (**B**) Pancreatic adenocarcinoma (T1), (**C**) Desmoplastic small round cell tumor of the peritoneum (T2), (**D**) Primary peritoneal carcinoma (T3), (**E**) Moderately differentiated adenocarcinoma of the large intestine (T4), (**F**) Mucinous carcinoma of the appendix (T6), (**G**) Squamous cell carcinoma of the anal canal (T7), (**H**) Pancreatic adenocarcinoma (T8), (**I**) Pancreatic neuroendocrine carcinoma (T9) and (**J**) Adenocarcinoma of the colon (T10). n=48. ****p<0.0001, ***p<0.001, **p<0.01 and *p<0.05 compared with control spheroids. Results are presented as mean ± SEM.

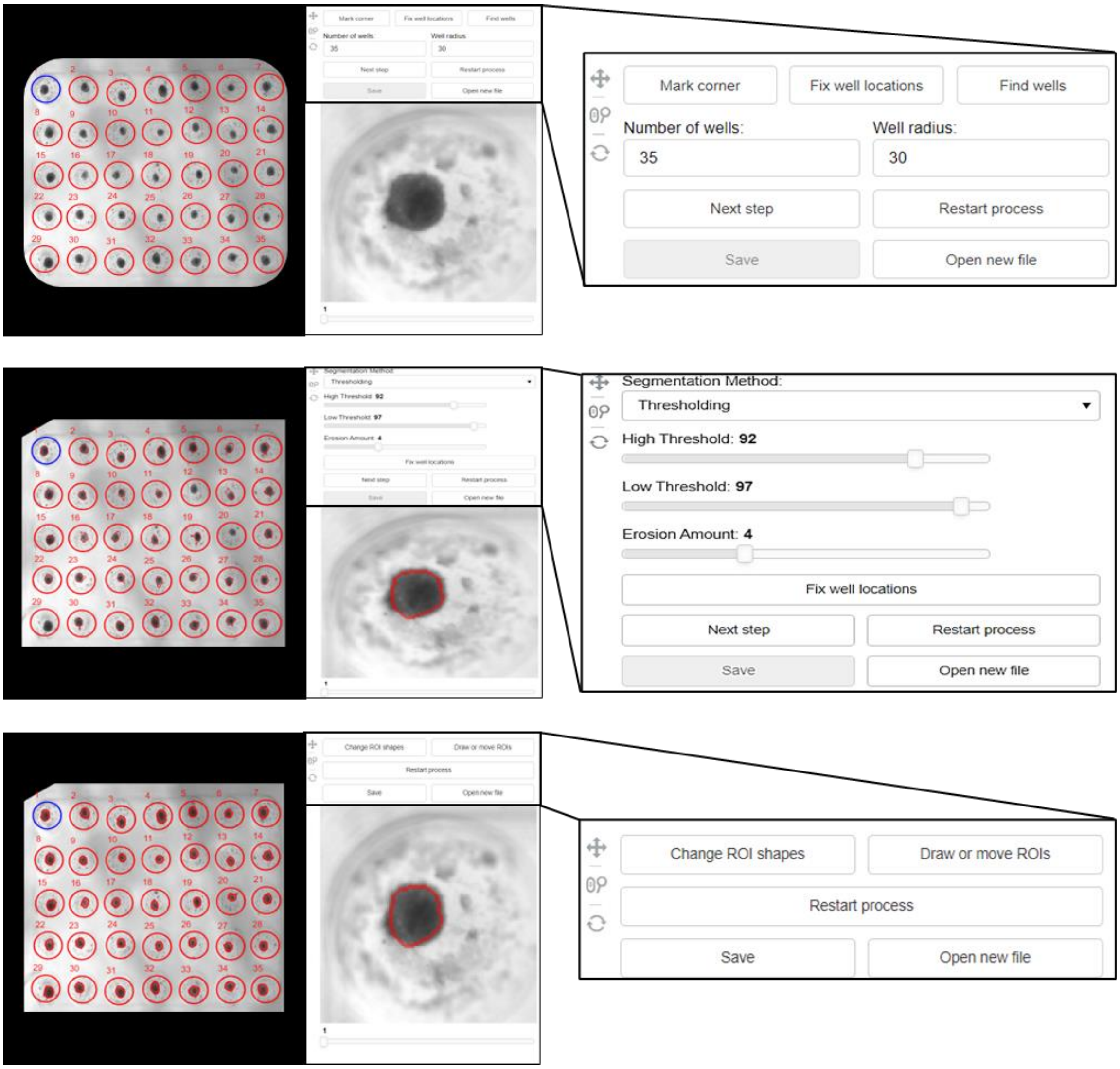

Fig. S7. Semi-automatic segmentation of spheroids. To make spheroid area measurements easier to calculate, semi-automatic segmentation techniques can be used. The code can be found at: https://github.com/friedmanroy/CIDR-cell-segment. In the first step, the number of wells in the current mold can be inserted. Secondly, the mold corner can be marked (shown as blue circle around corner well). Next, the locations of the wells can be accurately fixed (shown as red circles). The marking around the spheroids can then be inserted by adjusting the threshold and the amount of erosion/smoothness at the spheroids' periphery. The area marking can be adjusted next either by rearrangement of an existing marking or by drawing a new marking. Data can then be saved as an Excel file containing area markings in pixel or micron units and as images depicting the markings around the spheroids and wells.

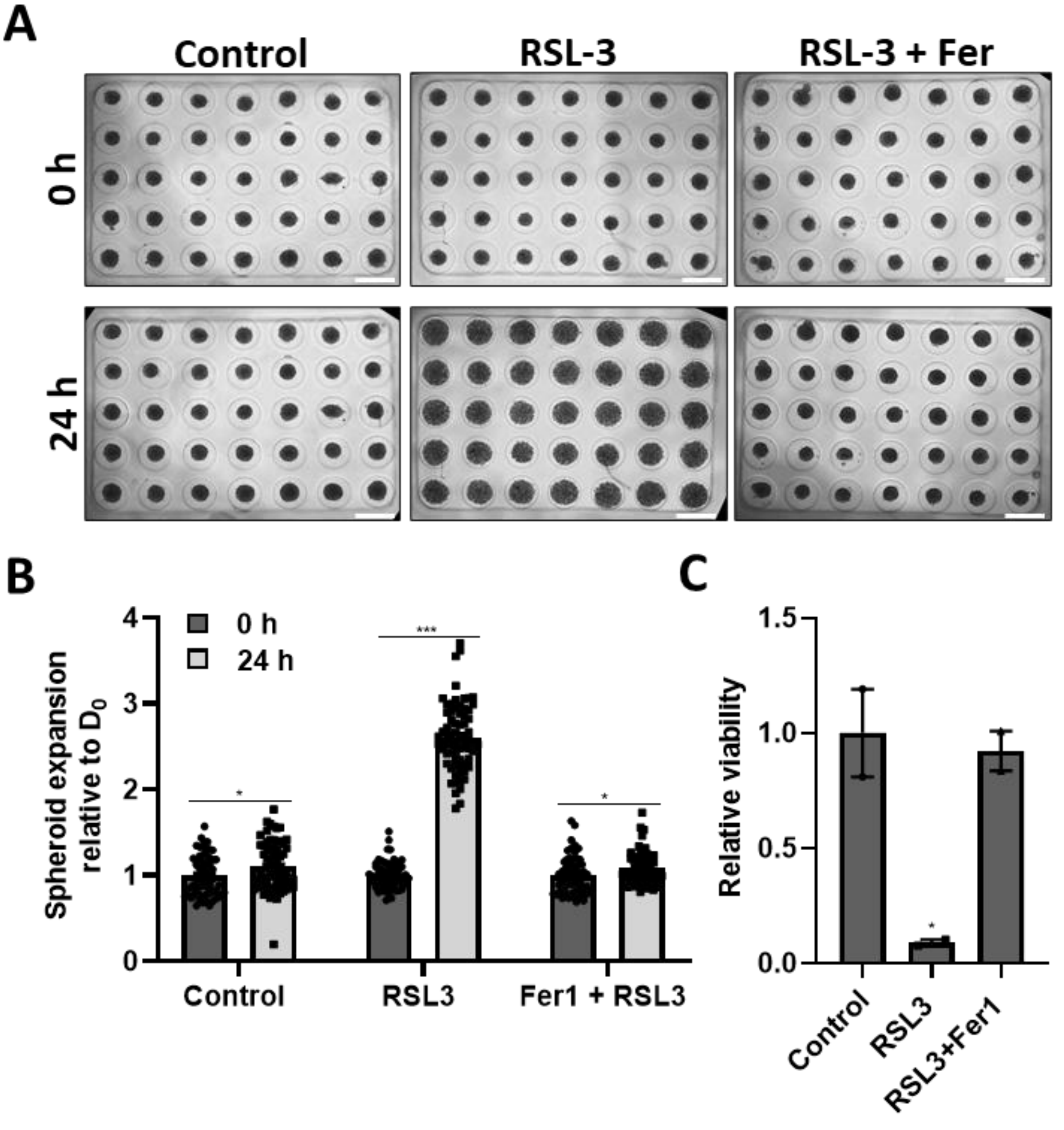

Fig. S8. Ferroptosis induced in BeWo spheroids results in significant area growth. (**A**) Images of BeWo spheroids 48 h after seeding at 5,000 cells/microwell (depicted as 0 h). After 48 h, 250 nM of RSL3 or 1,250 nM RSL3 and 0.5 µM Ferrostatin-1 (Fer1) were added for a 24 h incubation. Scale bar = 1 mm. (**B**) Analysis of spheroid area 48 h after seeding (depicted as 0 h) relative to spheroid area 24 h after the addition of treatment. n=62. (**C**) WST1 viability assay was performed on

BeWo spheroids 24 h after the addition of treatments. n=3. ***p<0.001 and *p<0.05 compared with control spheroids. Results are presented as mean ± SEM.

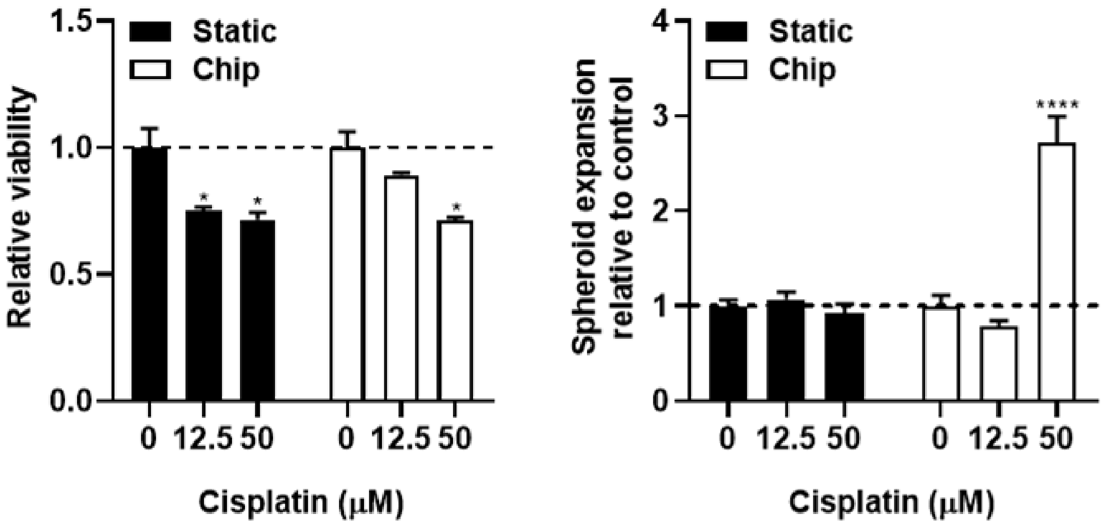

Fig. S9. BxPC-3 spheroids treated with Cisplatin in a "nut and bolt" chip. Cisplatin treatment was initiated 72 h after seeding at 8,000 BxPC-3 cells/spheroid in 4% agarose molds placed in the microfluidic device. Molds containing spheroids grown under static conditions were placed in a 96-well plate. A viability assay was performed on BxPC-3 spheroids 5 d after treatment initiation. n=3-6. Area analysis is depicted as the area expansion on day 5 relative to 72 h after seeding. Data shown were reanalyzed by comparing the response to a given treatment concentration to that under control conditions (no added treatment: set to 1, shown as a dotted line). n=14-35. ****p<0.0001 and *p<0.05 compared with control spheroids. Results are presented as mean ± SEM.
