# Supplementary material for: A fully 3D-printed versatile tumor-on-a-chip allows multi-drug screening and correlation with clinical outcomes for personalized medicine
Source: Commun Biol. 2023 Nov 13;6:1157. doi: 10.1038/s42003-023-05531-5 (PMC10643569; doi:10.1038/s42003-023-05531-5)
Supplement: Supplementary file 2 — Description of Additional Supplementary Files [file 42003_2023_5531_MOESM2_ESM.pdf]

Description of Additional Supplementary Files

File name: Movie S1
Description: Movie showing the "shaking test" of the hanging drops described in Figure 3 and Figure S3.

File name: Supplementary Data 1
Description: The source data behind the main graphs in the paper.
